# The gut metabolome in a cohort of pregnant and lactating women from Antioquia-Colombia

**DOI:** 10.3389/fmolb.2024.1250413

**Published:** 2024-05-13

**Authors:** Sara Londoño-Osorio, Lizeth Leon-Carreño, Mónica P. Cala, Laura Sierra-Zapata

**Affiliations:** ^1^ CIBIOP Research Group, School of Applied Sciences and Engineering, Universidad EAFIT, Medellín, Colombia; ^2^ MetCore–Metabolomics Core Facility, Vice-Presidency for Research, Universidad de Los Andes, Bogotá, Colombia

**Keywords:** gut microbiome, pregnancy, lactation, perinatal nutrition, untargeted metabolomics, molecular networking, gut metabolome

## Abstract

Nutrition during the perinatal period is an essential component of health and one that can severely impact the correct development of a human being and its overall condition, in all the subsequent stages of life. The availability of several compounds, mainly macronutrients and micronutrients, plays a key role in the balanced nutrition of both mother and baby and is a process with direct relation to the gut microbiome. Thus, we hereby refer to the set of small molecules derived from gut microbiome metabolism as the gut metabolome. These continuous processes occurring in the gut of a gestating or lactating mother related to microbial communities and nutrients, can be revealed by metabolomics. In this study, we explore for the first time the gut metabolome of pregnant and lactating women, from our region of Antioquia-Colombia, applying untargeted metabolomics by LC-QTOF-MS, and molecular networking. Regarding the gut metabolome composition of the cohort, we found, key metabolites that can be used as biomarkers of microbiome function, overall metabolic health, dietary intake, pharmacology, and lifestyle. In our cohort, pregnant women evidenced a significantly higher abundance of prostaglandins, alkaloids, corticosteroids, organosilicons, and natural toxins, while in lactating women, lipids stand out. Our results suggest that unveiling the metabolic phenotype of the gut microbiome of an individual, by untargeted metabolomics, allows a broad visualization of the chemical space present in this important niche and enables the recognition of influential indicators of the host’s health status and habits, especially of women during this significant perinatal period. This study constitutes the first evidence of the use of untargeted LC-QTOF-MS coupled with molecular networking analysis, of the gut microbiome in a Colombian cohort and establishes a methodology for finding relative abundances of key metabolites, with potential use in nutritional and physiological state assessments, for future personalized health and nutrition practices.

## Introduction

All nutrients come from the diet, and diet is one of the most important aspects impacting and modulating health and the gut microbiota. This ‘microbial’ organ within our guts, and the set of genes it contains, called the microbiome (El Hage et al., 2017) have been extensively studied over the last decade. Several of these studies, now published in prestigious journals, have uncovered that dysbiosis, or an imbalance of the intestinal microbial communities (microbiota) and the decrease in ecological diversity within the gut, are related to gastrointestinal, metabolic, and autoimmune diseases, mental disorders, and even some types of cancer (Derrien and Veiga, 2017; Deng et al., 2021; Zhao et al., 2021; Christovich and Luo, 2022; Horn et al., 2022). Since the gut microbiome has a crucial role in the absorption and metabolism of nutrients, both macro and micro, aiming for a balanced microbial community in the gut, helps maintain the host homeostasis, and builds the intestinal barrier (DAS & Nair, 2019). The presence or absence of specific microbial genera or species has been associated with multiple diseases, most of them, non-communicable ones, such as inflammatory bowel disease, diabetes, obesity, some types of cancer, Parkinson’s, and Alzheimer’s, among others (Novakovic et al., 2020; Zhang et al., 2020; Bardenhorst et al., 2023). Regarding micronutrient absorption, Hadadi and collaborators (2021) addressed the importance of the gut microbiome for maintaining the balance of the host vitamins and minerals. They also address the micronutrient-microbiome axis as a bidirectional entity, and according to other studies, several micronutrient deficiencies could be positively or negatively associated with the gut microbiota (Hadadi et al., 2021). Another study carried out by Maynard and Weinkove has revealed that certain host microbes, such as *C. elegans* and *E.coli,* play a role in the effective supplementation of micronutrients by the secretion of siderophores (iron and B12), or the uptake and conversion into more readily absorbable derivatives or micronutrients, such is the case of folic acid (Maynard and Weinkove, 2020). Moreover, Bielik and Kolisek (2021) reported the positive effect of probiotics on mineral absorption, stating they are promising due to their ability to modulate the composition and metabolism of the gut microbiota (Bielik and Kolisek, 2021).

On the other hand, the perinatal period is marked by hormonal, immunological, and—especially during the late stages of healthy pregnancies without complications—by inflammatory changes that alter the function and bacterial composition of the mother’s gut (Mandal et al., 2016). Estrogen and progesterone also impact this composition through their effect on bacterial metabolism and the increase in abundance of pathogenic bacteria (Edwards et al., 2017). It is also known that the gut microbiota contributes to the regulation of glucose metabolism in pregnancy (Brantsæter et al., 2011). For example, the abundance of the genus *Collinsella* sp. Is positively correlated with circulating insulin, and low dietary fiber intake was associated with a gut microbiota favoring lactate fermentation, while high fiber intake promotes short-chain fatty acid-producing bacteria (Fu et al., 2022). Related to this, low dietary fiber may enable the overgrowth of *Collinsella* sp. and alter the overall fermentation pattern in gut microbiota (Gomez-Arango et al., 2018). This suggests that dietary choices during pregnancy can modify the nutritional ecology of the gut microbiota. Besides, in a study conducted on pregnant women, it was shown that there are significant differences in the relative abundance of several genera in women on a vegetarian diet, specifically a reduction in *Collinsella* sp., *Holdemania* sp., and an increase in the relative abundances of *Roseburia* sp. and Lachnospiraceae sp. (Barrett et al., 2018). The most recent research on gut microbiome during the perinatal period in mice shows that the characteristic microbiota of the third trimester of pregnancy, increases weight gain, insulin resistance, and a greater inflammatory response when transferred to germ-free mice (Koren et al., 2012). Studies in other populations different from the American and European ones, such as those from Latin America, the Caribbean, Asia and African, or from women and children’s cohorts, are urgently needed as well as their underlying data (Magne et al., 2016), in order to properly acknowledge the gut microbiome in world-population scale, and be able to develop solutions to improve the health status of the groups belonging to these communities, in need of tools for this purpose.

In the quest for the characterization of generalizable traits of the gut microbiome, metabolomics has appeared as one of the most useful techniques to study it, being defined as a comprehensive analysis of all metabolites in a biological system with their proper identification and quantification (Fiehn, 2002), and is recognized as a powerful top-down systems biology approach, for understanding the genetics-environment-health paradigm and identifying clinically relevant biomarkers (Moco et al., 2013). Metabolomics studies within the gut, which we name here the gut metabolome, have been increasing in the last years due to the strong relationship found between some gut microbiome metabolic pathways and diseases, especially non-communicable ones, and due to the involvement of the gut microbiota in several biochemical functions directly associated with perturbations that can lead to the development of diseases (De Preter et al., 2015). Moreover, the identification and relative quantification of metabolites in these environments can point out lifestyle and dietary habits, and nutrient balance in the gut, which in turn, allows the highlighting of specific disease predispositions (Vernocchi et al., 2012), such as a mineral or vitamin deficiency of (Lai et al., 2022; Wan et al., 2022), an excess of an inflammatory molecule (Zhang et al., 2021), among others. Metabolomics is a technique that can be performed over different biological matrices such as cells, tissues, stool samples, and biofluids such as plasma, saliva, urine, and blood. The sample selection will always depend on the research or clinical question, but biofluids are typically used to identify biomarkers, whereas tissues and cells are used to investigate mechanisms associated with the pathophysiological process (Chetwynd et al., 2017). Regarding human stool samples, which reflect the gut metabolome, most of the published research has focused on characterizing its complex bacterial composition using next-generation microbial DNA sequencing and sophisticated metagenomic techniques. However, a growing number of microbiome researchers are recognizing that considerable information could be gained by using a more integrative approach that also includes comprehensive fecal metabolite analysis (Karu et al., 2018; Haffner et al., 2022).

One of the techniques widely used to study the gut (fecal) metabolome, is liquid chromatography coupled to mass spectrometry (LC-MS), which does not usually include derivatization steps. The technique can be performed in a targeted or untargeted mode, depending on the experimental design, and multiple approaches can be taken to analyze the raw data, thus allowing the recognition of multiple chemical families and the greater elucidation of the chemical space, phenotype, and nutrients composition of the gut. In this research, we used classical molecular networking and untargeted metabolomics to make a pilot and first approach toward the characterization of the chemical space of the gut microbiota (gut metabolome) of women from Antioquia, Colombia. These women conform to a pilot cohort (n = 23) of pregnant, 7) lactating 9), and reproductive-age women 7) acting as controls. By using LC-QTOF-MS/MS metabolomic techniques and data analysis, we aimed at the identification and quantification of several compounds of nutritional importance for the baby’s appropriate development, which are supplied by the mother during these fundamental stages of pregnancy and lactation. As stated above, macronutrients, micronutrients, and derived metabolites play a key role in the balanced nutrition of both mother and baby, and both are intrinsically related to the gut microbiome. Thus, with this pilot study, we wish to contribute to the maternal nutritional body of knowledge in our area of the world since to date, there are no published studies that explore the chemical diversity of the Colombian female population during the mentioned stages, despite these being key interventional periods for nutrition. It is our wish that the knowledge derived from this pilot study and its validation in larger cohorts can help avoid future developmental complexities in an individual during later stages of their lives also avoiding future health complications. Thus, the relevance of this kind of pilot study and as mentioned earlier, the further validation of its preliminary results in larger cohorts is evident, to broaden our knowledge of the gut microbiome chemical space and phenotype in the populations in Colombia, Latin America, and the Caribbean.

## Materials and methods

### Study cohort and sample collection

**FIGURE 1:**
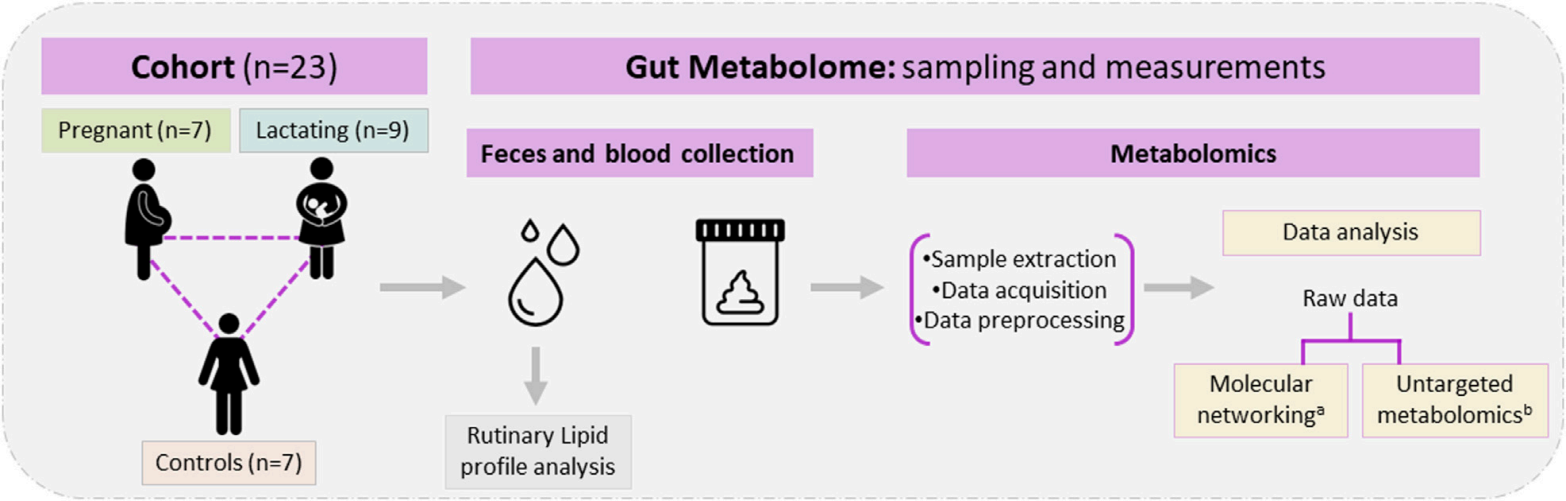
Study design, cohort composition, samples used, and metabolomic analysis. ^**a**^ **Classical molecular network was performed using GNPS and Cytoscape.** ^**b**^ **Untargeted metabolomics used MetaboAnalyst 5.0, MATLAB R2021b, and CEU mass mediator, the libraries Kegg, HMDB, METLIN, lipid maps, and MS-DIAL 4,80 software for metabolite identification.**

A group of twenty-three women volunteers, from Antioquia, Colombia between 23 and 35 years old were enrolled in the study, between August 2020 and May 2021. Nine of them were lactating, seven were pregnant and seven were control group (non-pregnant or lactating women) of reproductive age (Figure 1). Both pregnant and lactating women were enrolled since they complied with a healthy pregnancy/lactation stage, without complications. Average values of the different variables measured for each group of the cohort are detailed in Table 1, as well as detailed information for each volunteer (age, height, weight, pregnancy or *postpartum* week, lipid profile) which was saved as correlated metadata for the study. As inclusion criteria, the selected cohort must declare non-consumption of antibiotics in the past 6 months before the sample collection. Two different samples were taken from each volunteer, a blood sample was collected in collaboration with Abad Laboratory, to measure the lipid profile (low-density lipoprotein (LDL), high-density lipoprotein (HDL), triglycerides, and total cholesterol), and a stool sample was provided. This last sample was processed at Universidad EAFIT, within the next 24 h of collection, in an anaerobic chamber (Vinyl Anaerobic Chamber Type B from Coy Laboratory Products). Briefly, 200 mg of it was homogenized in 1 mL pH 7.2 buffer solution (0.05% K2HPO4, 0.05% KH2PO4, 0.05% MgSO4 x 7H2O, 0.0005% FeSO4 x 7H2O, 0.005% (NH4)2SO4, 0.1% cysteine, 0.001% resazurin, and 20% glycerol) (Hayashi et al., 2002) and stored at −80°C for any subsequent use.

**TABLE 1:** Characteristics of the study cohort. Values of total cholesterol, LDL, HDL, and triglycerides are presented in (mg/dL) units.

	Lactating	Pregnant	Control
**Number of volunteers**	9	7	7
**Age (years)**	32.9 ± 2	28.6 ± 4.4	30.4 ± 10.7
**BMI**	22.5 ± 2.1	25.7 ± 67	21.4 ± 1.2
**Total Cholesterol**	215.67 ± 55.1	243 ± 50.4^*^	175.6 ± 32.8*
**LDL**	129.59 ± 51.8	127.9 ± 39.4	101.5 ± 29.2
**HDL**	62.3 ± 6.8*	78.9 ± 15.6*	60.5 ± 12.1*
**Triglycerides**	100.8 ± 49.8*	181 ± 45.1*	68.2 ± 17.3*
**Gestational week**	0	27.29 ± 3.9	0
***Postartum* week**	13.3 ± 15.9	0	0

Super index *: Triglycerides and HDL (*p*-value = 0.01 and 0.03 respectively) were statistically different in pregnant women compared to lactating women. Triglycerides had a (*p*-value = 0.01) for lactating women vs. the control group. Triglycerides, HDL, and total.cholesterol (*p*-value = 0.002, 0.03, and 0.04 respectively) were statistically significant between the pregnant and control group.

## Sample treatment

Frozen Stool samples were weighted, lyophilized at −80°C for 72 h, and weighed again to determine the removed water content percentage. Then, nitrogen gas was injected for 10 min into each sample to guarantee an inert environment. For extraction, 60 mg of each lyophilized sample was mixed with 300 µL of MeOH and vortex-mixed for 5 min. Subsequently, samples were taken to an ultrasound for 30 min and vortex-mixed again for 5 min. Finally, samples were centrifuged at 180,00x g, 4°C for 15 min and 100 µL of the extract was used for the analysis by LC-QTOF-MS (Cheng et al., 2020).

### Metabolomic analysis

#### Data acquisition for untargeted metabolomics and molecular networking using RP-LC/MS and HILIC-LC/MS

Metabolomics data from fecal samples were acquired using an Agilent Technologies 1,260 Liquid Chromatography system coupled to a 6545 Q-TOF quadrupole time-of-flight mass analyzer with electrospray ionization. For the reversed-phase, 2 µL of the sample was injected into a C18 column (InfinityLab Poroshell 120-EC 100 × 2.1 mm, 1.9 µm) at 40°C. The mobile phases used for elution were composed of 0.1% (v/v) formic acid in Milli-Q water (Phase A) and 0.1% (v/v) formic acid in acetonitrile (Phase B) pumped at 0.4 mL/min with a gradient starting at 5% B, increased at 96% B in 15 min and kept there 1 min and then, at 16.1 min, going back to the initial conditions until 20 min. Detection by mass spectrometry was performed in positive ESI mode in full scan and autoMS/MS from 50 to 1,100 m*/z* and 20eV. Throughout the analysis, two reference masses were used for mass correction: *m/z* 121.0509 [C_5_H_4_N_4_ +H]^+^, and *m/z* 922.0098 [C_18_H_18_O_6_N_3_P_3_F_24_ + H]^+^, corresponding to protonated purine and protonated hexakis, respectively.

For hydrophilic interaction chromatography, 5 µL of the sample was injected into a HILIC-Z (InfinityLab Poroshell 100 × 2.1 mm, 1.9 µm) column, which was thermostated at 30°C. The elution gradient was composed of 10% (200 mM ammonium formate pH 3): 90% H_2_O (Phase A) and 10% (200 mM ammonium formate pH 3): 90% ACN (Phase B) with a constant flow of 0.5 mL/min. The chromatography gradient started at 100% of phase B and decreased to 70% B in 10 min. The starting condition was returned by minute 11 and kept there for 5 min for re-equilibration time. Data were collected in negative mode operated in full scan and MS/MS mode at 20 eV from 50 to 1,100 m*/z*.

#### Data processing and analysis for untargeted metabolomic analysis approach

The full scan raw data from RP-LC/MS and HILIC-LC/MS was processed using Agilent MassHunter Profinder Software B.08.00. The software uses the Molecular Feature Extraction (MFE) technique and Recursive Feature Extraction algorithms for noise reduction, feature deconvolution, and alignment. The data matrices from each platform were filtered by presence and reproducibility, keeping only the metabolites detected in at least 80% of all stool samples and using a threshold of 20% based on the coefficient of variation (CV) of metabolite levels in the quality controls (QCs). Differences among the groups were explored using both multivariate (MVA) and univariate (UVA) statistical analyses. For MVA, a partial least-squares discriminant analysis PLS-DA model was used for sample classification and to detect differences between the groups using MetaboAnalyst 5.0 (https://www.metaboanalyst.ca/MetaboAnalyst/ModuleView.xhtml). Metabolites with variable importance in projection (VIP) ≥ 1 and a jackknifing confidence interval that did not include zero were considered statistically significant from the PLS-DA models. The univariate analysis employed in this study used the Mann-Whitney *U* test in MATLAB R2021b to evaluate the significant differences between each metabolite (*p-*value < 0.05) in the following comparisons: Lactating vs Control, Pregnant vs Lactating, and Pregnant vs Control.

### Metabolite identification

To annotate statistically significant metabolites, the CEU Mass Mediator tool (http://ceumass.eps.uspceu.es/) was used, which matches metabolites with libraries, in addition to analyzing their correspondence with the mass spectral library and the generated molecular formula. The databases Kegg (http://genome.jp/keg), HMDB (http://hmdb.ca), METLIN (http://metlin.scripps.edu), and Lipid MAPS (http://lipidmaps.org), as well as the software MS-DIAL 4.80 (http://prime.psc.riken.jp/compms/msdial/main.html) and Agilent MassHunter qualitative analysis software, were also utilized for this purpose. The identification level assigned to each compound was according to the Metabolomics Standards Initiative (MSI) by Fiehn (Sumner et al., 2007) where level 1 corresponds to the metabolites identified by reference standard, level 2 to those that have MS/MS spectrum match and molecular formula, level 3 with unequivocal molecular formula, and level 4 only with *m/z* database match.

#### Data processing and analysis for molecular networking approach

For classical molecular networking, raw data (.d files) obtained from the data acquisition with C18 and HILIC columns, were converted into (.mzXML) format using MSconverGUI (Holman et al., 2014). Once the data were confirmed to be reproducible and a separation between groups was observed, the datasets were uploaded to GNPS web platform GNPS–Analyze, Connect, and Network with your Mass Spectrometry Data (ucsd.edu) (Wang et al., 2016) under de massive code MSV000088880 MassIVE Dataset Summary (ucsd.edu) for C18 data, and MSV000089161 MassIVE Dataset Summary (ucsd.edu) for HILIC data. Two classical molecular networks were built to visualize the features present in the samples’ chemical space, and clustered by chemical families. In a second layer of information, each feature was classified by color, as being part of either one cohort group, two of them, or being a shared feature across the three groups in the study. Each group (lactating, pregnant, control) had seven volunteers meaning seven different datasets that act as replicates of the chemical space of the said physiological state; we included a fourth group which consisted of a mix of pure standards of dietary choline derivatives as a control for this specific micronutrient, highly important during pregnancy and lactation. Several of choline’s biochemical route derivatives in the gut microbiome were included, these being acetylcholine, betaine, phosphatidylcholine, choline chloride, and trimethylamine. The network parameters set in the GNPS platform were (Min pairs cosine: 0.75, Min fragmented ions: 0.6, Min matched peaks: 6, Cluster size: 2, Analog search: do search). Then, the generated molecular networks were exported to Cytoscape (Ideker, 2003), following manual annotation and curation of the clusters.

### Network curation and annotation

This procedure was followed as proposed by Sierra-Zapata et al. (2020). The total features table was exported from Cytoscape as (.csv) file to analyze the abundances of each feature based on the spectral count and the identification provided by the platform (GNPS) for each feature*.* A query was used to extract the nodes information of each sub-network (Supplementary Tables S1, S2) and based on the library hit found for a feature through GNPS, we assigned a chemical family name to each sub-network, by looking at the metabolite’s functionality in PubChem. When non-conclusive, a search in ChemSpider and the Human metabolome database was done as well. In the cases where a unique node from the sub-network was annotated, the entire sub-network was labeled by the same chemical family, and when different nodes were identified, the family name was given following the functionality that grouped all of them. This is done in accordance with the algorithm of GNPS, where a single node’s annotation, can be propagated to its neighboring nodes connected by edges, given structural similarity clustering (Wang et al., 2016). In Cystoscope, the nodes were colored according to their presence in each group of the cohort: light blue for the lactating group, dark green for the pregnant group, orange for the control group, red for standard metabolites, and purple for the group of metabolites present in both lactating and pregnant women. This network was exported in (.pdf) format with the precursor mass available as a label on the nodes, and the chemical family was then added as a circle grouping the cluster of nodes. Given the family name and its abundance (in numbers of spectra) among the treatments, the relative abundance for each chemical family was calculated in each group of the cohort to see any statistical difference (Supplementary Material S1, S2). Also, a PCA was performed into MetaboAnalyst using the raw data to visualize any clustering of the chemical space of the cohort’s groups.

## Results

### Untargeted metabolomics analysis by RP-LC/MS and HILIC-LC/MS

**FIGURE 2:**
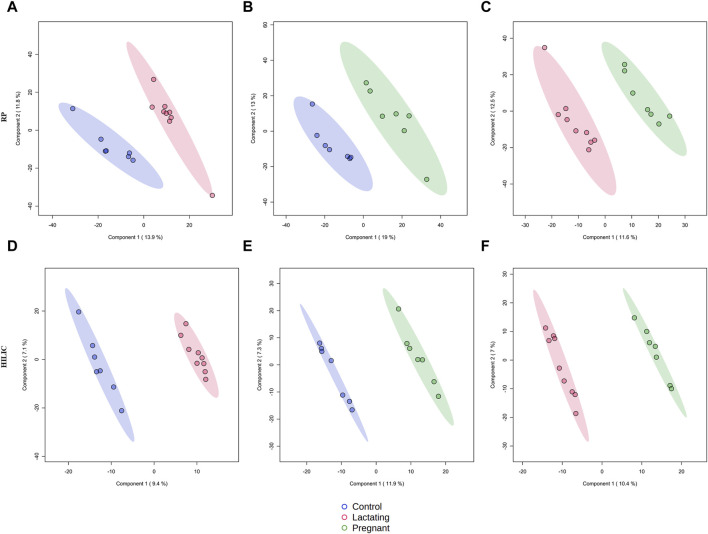
Supervised PLS-DA models for metabolomics by reverse-phase (RP) in positive mode and Hydrophilic interaction chromatography (HILIC) in negative mode. **(A)** *R* ^2^:0.95404, Q^2^:0.21976; **(B)** *R* ^2^: 0.956, Q^2^: 0.29733; **(C)** *R* ^2^: 0.9692, Q^2^: 0.12499; **(D)** *R* ^2^: 0.99479, Q^2^: 0.37436; **(E)** *R* ^2^: 0.99663, Q^2^: 0.27773; **(F)** *R* ^2^: 0.99622, Q^2^: 0.40789.

The cohort of volunteers and their characteristics, from where the data were obtained, can be revised in Figure 1 and Supplementary Datasheet S1. First, untargeted metabolomics analysis of the data acquired from the volunteers’ samples, according to the methods described above, was performed. A multivariate analysis (MVA) was made using PLS-DA (Figure 2) to compare data from Lactating vs. Control, Pregnant vs. Control, and Lactating vs. Pregnant treatments. The PLS-DA model shows values of *R* ^2^ ranging from 0.95 to 0.99 and Q^2^ from 0.21 to 0.40 indicating a clear separation between the comparison of features acquired by RP-LC/MS and HILIC-LC/MS and thus can be considered a good feature selector model. Then, a univariate analysis (UVA) was conducted to identify the differential metabolites between the proposed comparisons. A total of 200 differential molecular features were identified in both platforms through UVA and MVA analyses, considering those with a *p-value < 0.05* or VIP>1. Among them, 85 metabolites were statistically different when comparing lactating and control groups, 67 metabolites when comparing pregnant and control groups, and 48 metabolites between pregnant and lactating groups. Supplementary Table S3 shows the metabolites that were detected as up or downregulated among the groups of the study, including the significance metrics provided by the MVA and UVA (VIP and *p-*value).

**FIGURE 3:**
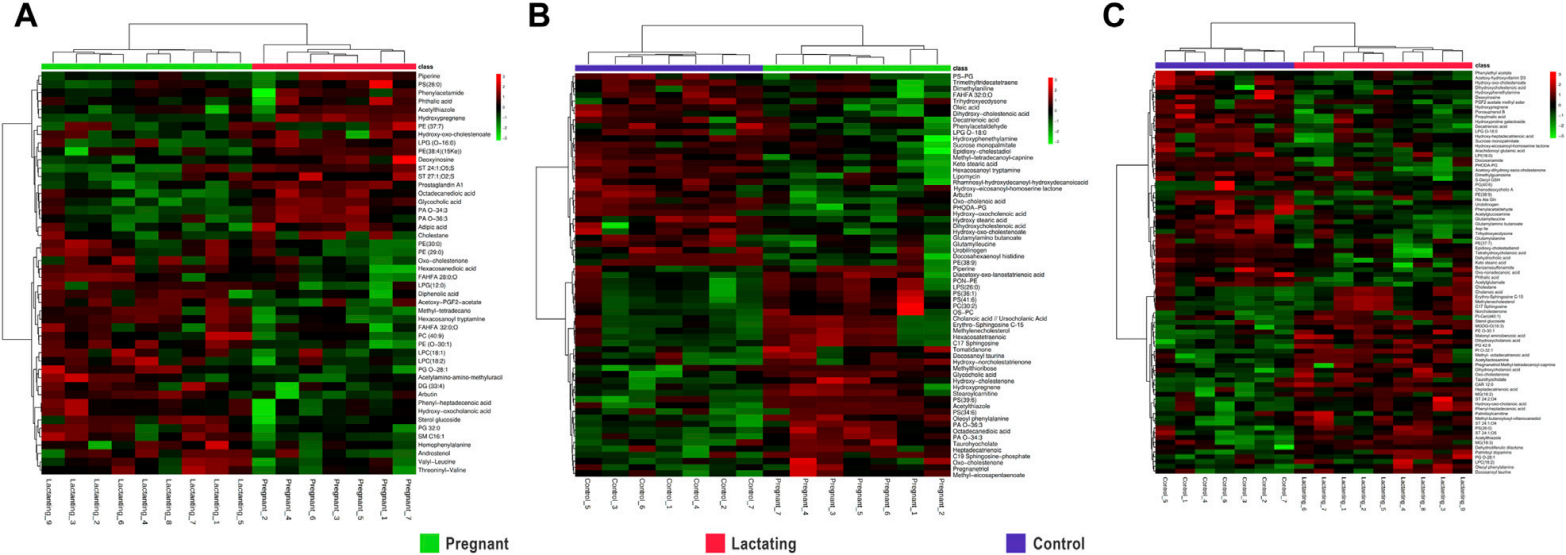
Hierarchical Clustering Heatmaps of metabolome data **(A)**. Pregnant vs. Lactating **(B)**. Pregnant vs. Control **(C)**. Lactating vs. control.

For pregnant women compared to lactating ones, it has been found that piperine, benzenoids, hydroxypregnene, glycerophosphoserines, glycerophosphates, deoxyinosine, prostaglandins, biotin, and steroids (Figure 3A, Supplementary Table S3) Pregnant vs. Lactating) were upregulated. Specifically, hydroxypregnene, deoxyinosine, prostaglandins, and steroids were detected as diminished in lactating women vs controls, thus being differentially detected in the guts of pregnant, lactating, and women of reproductive age.

Then, when comparing pregnant women with non-pregnant nor lactating controls (Figure 3B) increased levels of alkaloids, bile acids, carbohydrates, corticosteroid hormones, some fatty acids, glycerophosphocholines, glycerophosphoserines, glycerophosphates, sphingolipids, and sterols were found. Meanwhile, glycerophosphoglycerols, steroids, and 53% of the total fatty acids found showed a decrease in the pregnant group. Specifically, glycerophosphocholines and glycerophosphoserines, corticosteroid hormones, bile acids, fatty acids, carbohydrates, and sterols are increased both in lactating and pregnant women gut metabolome when compared to control women in the cohort, as found by this methodology of untargeted metabolomics.

Importantly, for lactating women compared to non-pregnant nor lactating women of reproductive age, it has been found that amines, phthalic acid, urobilinogen, acetylglucosamine, corticosteroid hormones such as hydroxypregnene, fatty amides, glycerophosphoglycerols, glycerophosphoethanolamines, glycerophosphoinositol, prostaglandins, peptides and proteins, polyketides, steroids, and vitamin D were mostly downregulated in a range of 0.1 to 0.7 fold change (Figure 3C; Lactating vs. Control). On the other hand, in the lactating group, there were also notable upregulations compared to controls observed in various chemical families, such as bile acids, carnitines, ceramides, glycerolipids, glycerophosphocholines, glycerophosphoserines, and palmitoyl dopamine. Furthermore, amino acids and derivates showed a 40% increase, as did carbohydrates (50%), benzoic acids (67%), fatty acids (63%), corticosteroid hormones (67%), and sterols (80%).

### Chemical space defined by molecular networking

**FIGURE 4:**
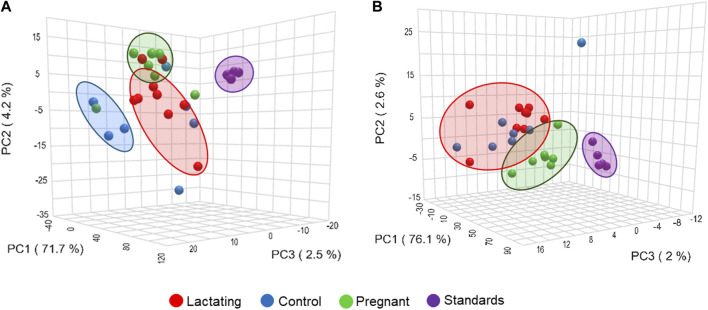
Principal component analysis (PCA) from LC-MS/MS raw data, **(A)** using C18 column and **(B)** HILIC column.

**FIGURE 5:**
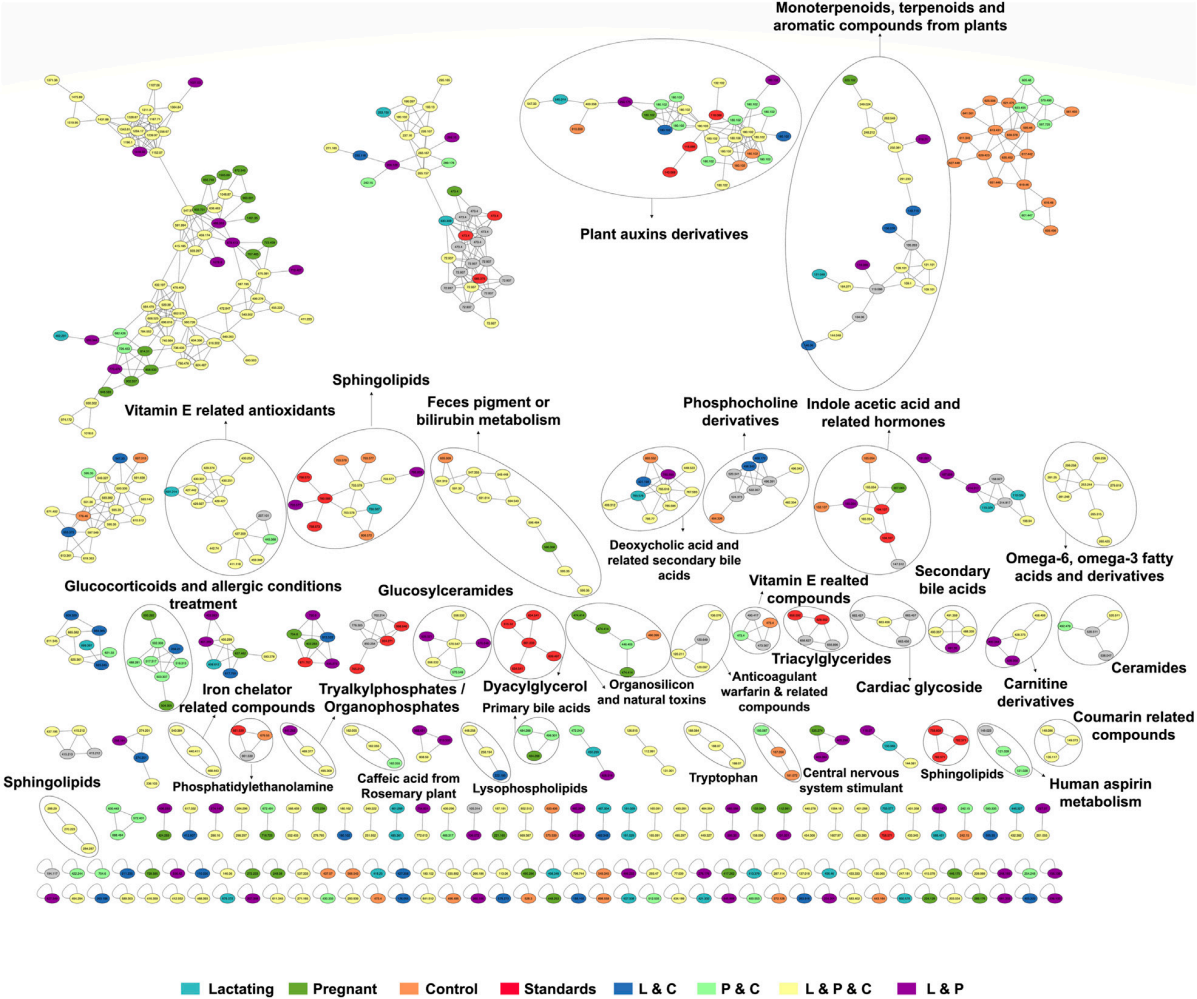
The molecular network created in GNPS for LC-MS/MS data acquired using the C18 column. Each node represents a single metabolite precursor mass (feature) and the color indicates the physiological state where each metabolite was found. Lactating (light blue), pregnant (green), control (orange), lactating and pregnant (purple), lactating and control (dark blue), pregnant and control (light green), lactating, pregnant and control (yellow), and the standards (red). Grey nodes denote the confluence of that feature in all groups within the study.

Before analyzing the data by molecular networking, a principal component analysis was performed by MetaboAnalyst (Xia et al., 2009), evidencing that for the HILIC platform, samples from the pregnant group clustered together and correlated (Figure 4B), separating themselves from the other cluster of control and lactating groups of volunteers. However, in the analysis by RP-LC grouping is not as evident as in the data obtained by HILIC, although a distinction is still observed between the volunteers in each group (control vs lactating vs pregnant ones, Figure 4A). When running the classical molecular network at the GNPS platform, we obtained 382 annotated metabolites (nodes or features) out of 1,583 (24% of the chemical space identified), for the C18 column, and 118 out of 465 (∼25% of the chemical space identified) for the HILIC column. A chemical family was assigned as the name to each sub-network that had at least one annotated metabolite, getting a total of 32 chemical families for the C18 column (Figure 5), and the relative abundance compared across groups of the study of the most biologically significant of them (20) is shown in (Figure 7A).

Among the chemical families identified, we observed the following in concordance with the untargeted metabolomics approach (results presented in the section above): glucosylceramides, sphingolipids, bilirubin metabolism, phosphocholine and derivatives, indole acetic acid and related hormones, glucosyIceramides, diacylglycerol, primary bile acids, amino acid (tryptophan), carnitine derivatives, omega-6, omega-3 fatty acids and derivatives. Of these, carnitine derivatives, ceramides, lysophospholipids, phosphocholine derivatives, secondary bile acids, and tryptophan were in higher abundance in lactating women. Nevertheless, we also observed a larger identification of phytonutrients or plant-derived metabolites in the gut metabolome of the cohort such as monoterpenoids, terpenoids, and aromatic compounds from plants, vitamin E related compounds, coumarin-related compounds, caffeic acid and from rosemary plant; as well as pharmaceutical molecules (glucocorticoids and allergic conditions treatment, human aspirin metabolism, cardiac glycoside, anticoagulant warfarin) when using this molecular networking untargeted metabolomics approach. We also detected compounds with a broad classification as organosilicons and other natural toxins, in the gut metabolome of the cohort, in a significantly higher abundance in control women (of reproductive age).

**FIGURE 6:**
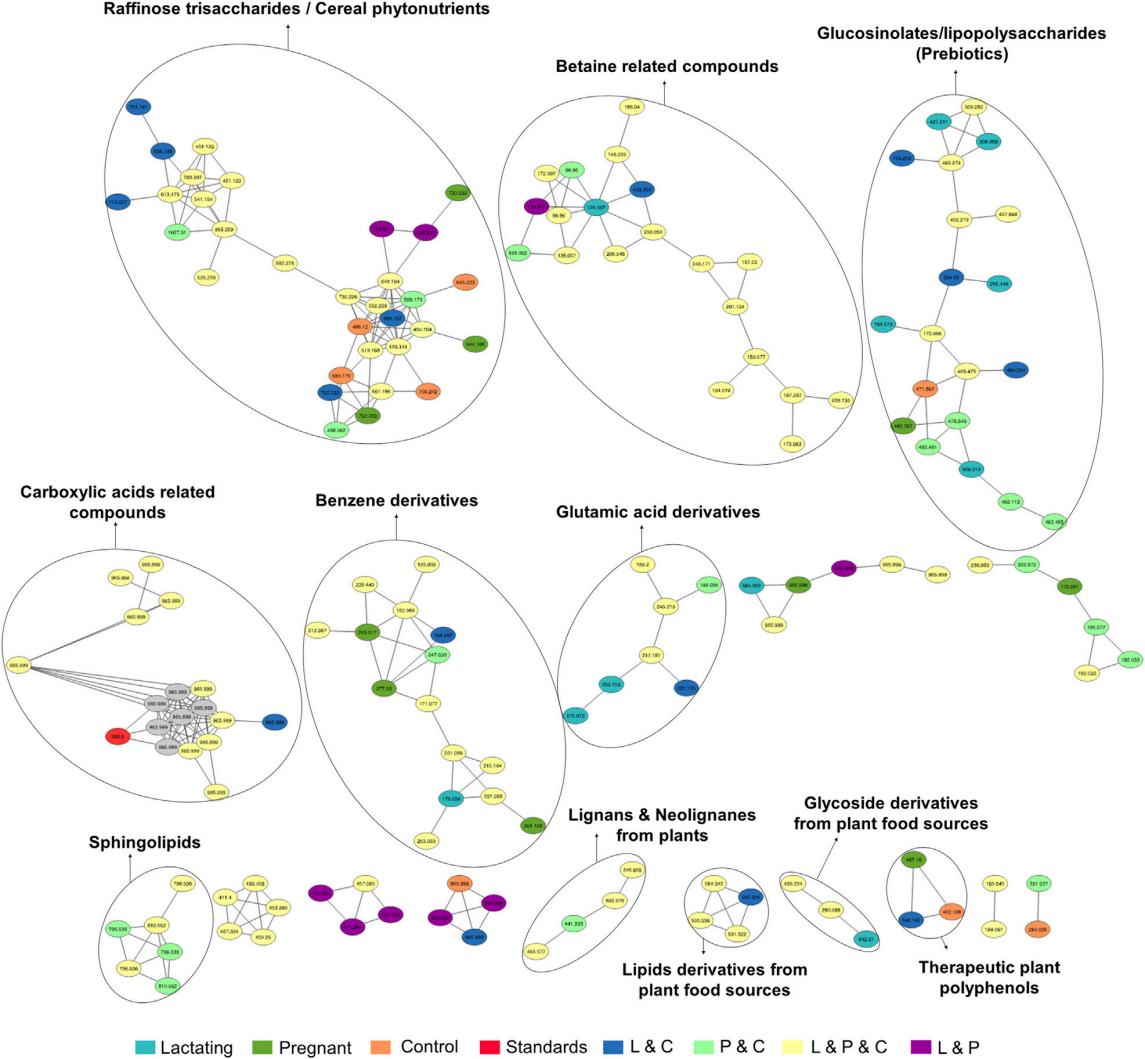
Molecular network created in GNPS for LC-MS/MS data acquired using HILIC column. Each node represents a single metabolite mass and the color indicates the physiological state where each metabolite was found. Lactating (light blue), pregnant (green), control (orange), lactating and pregnant (purple), lactating and control (dark blue), pregnant and control (light green), lactating, pregnant and control (yellow), and the standards (red). Grey nodes denote the confluence of that feature in all groups within the study.

**FIGURE 7:**
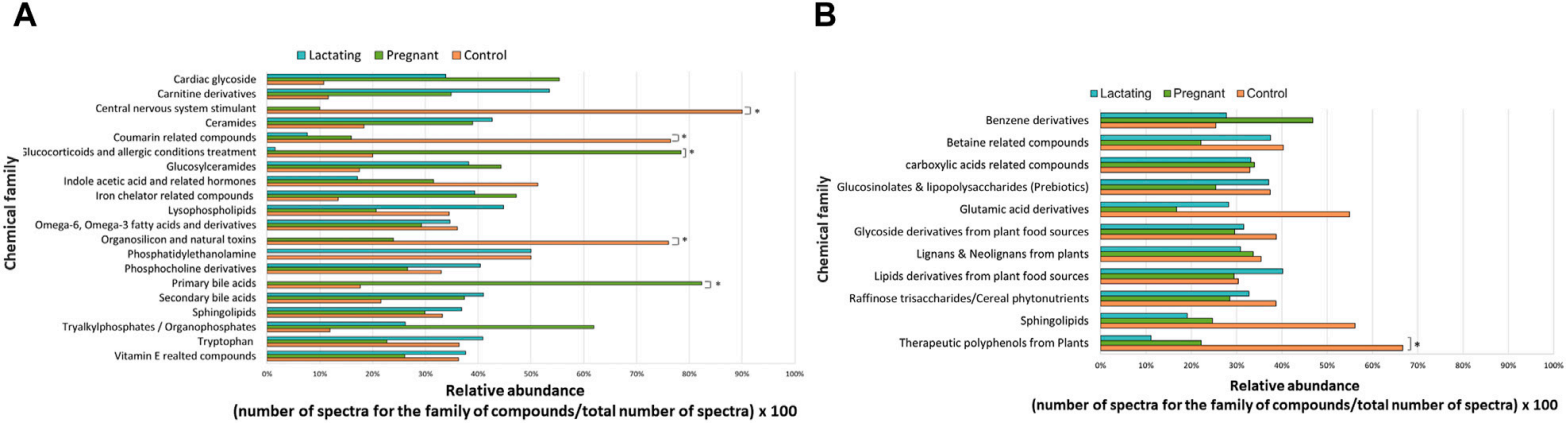
Relative abundance of the chemical families among the groups, detected by C18 **(A)** and HILIC **(B)** column. Asterisks represent those chemical families that are significantly different between groups (fold change ratio >1.8).

For the HILIC column, 11 chemical families were annotated (Figure 6) and the relative abundances of all of them are shown in (Figure 7B). The HILIC column, as a method able to detect polar compounds, allowed us to identify the following chemical families: betaine, phytonutrients as lignans and neolignans from plants, gIycoside and lipids derivatives from plant food sources, plant polyphenols, raffinose trisaccharides, glucosinolates and lipopolysaccharides which are proven prebiotic compounds (Zhang et al., 2022), glutamic acid derivatives, benzene derivatives, sphingolipids.

## Discussion

The gut microbiome of humans is estimated to comprise around 45 million non-redundant genes (Sender et al., 2016; Tierney et al., 2019). When compared to the human genome and its approximately 20.000 genes, the microbiota exceeds this capacity more than 1,000 times, evidencing its profound potential to influence the biochemical environment of the host (Lee-Sarwar et al., 2020). More surprisingly, the Human Microbiome Project discovery of metabolic pathway abundances in the gut is relatively consistent across populations, while taxonomic composition varies between individuals. This denotes that a core set of conserved pathways is associated with microbial genes, but their abundance varies depending on the taxonomic composition of this environment (Huttenhower et al., 2012). Besides, microbial functions are closely reflected by the composition of the metabolome, or better said, the collection of small molecules present in a sample. Although the human body houses many discrete microbiomes and metabolomes, the gut is taxonomically the most diverse and largest site (Thursby and Juge, 2017). Gathering the ideas exposed before, gut metabolome studies are now being considered the next Frontier to unveil the gut microbiome and are becoming prevalent in studies concerning this so-called organ, since they reflect the phenotype of the individual and thus, provide a more accurate perspective of the biochemical and metabolic processes taking place in this environment.

In this respect, our study offers various conclusions, some of them new and others reinforcing previous evidence from the scientific community on the gut microbiome and its associated metabolome. First, the gut metabolome or chemical space associated with the gut is a rich environment where important biomarkers of health can be detected (Figures 3–7). This is important in the way that the same metabolites are not always detected in serum metabolomics (Dhakan et al., 2019; Wen et al., 2020). Second, although our cohort is small (n = 23), the gut metabolome of pregnant, lactating, and women of reproductive age from our region of Antioquia (Colombia), evidences structural differences between groups both in its composition and relative abundance, denoting a plausible different core composition of microbial and host metabolism (Figures 2–6). In the case of microbial metabolism, these differences can be attributed to the differential taxonomic communities associated with each group of women according to their physiological state, as stated before (Koren et al., 2012). Specifically in this study, our findings suggest that, for the physiological stages of pregnancy and lactation, metabolites related to fats mobilization and membrane formation such as glycerophosphocholines, glycerophosphoserines, and fatty acids; hormones (corticosteroids); bile acids; carbohydrates and sterols are increased, being significantly overexpressed both in lactating and pregnant women’s gut metabolome (Figures 3–7). This result is expected since, specifically for lipids metabolism, previously published findings reporting multiple physiological changes that occur in healthy, gestating women, which contribute to the alterations in lipid profiles, mainly to support the developing fetus to whom cholesterol and essential fatty acids are essential for normal development (Wild and Feingold, 2000). Also, larger doses of foods rich in healthy fats are needed to meet the metabolic demand, especially for the nutrient choline, which is highly available in fats (Zeisel, 2013). Our findings, also correlate with a transformation of the gut microbiota into a proinflammatory immune state as pregnancy progresses (Koren et al., 2012; Trevisanuto et al., 2013), since prostaglandins, a main biomarker of both the promotion and resolution of inflammation (Ricciotti and Fitzgerald, 2011), are increased in this last group (Figure 3A). Also, corticosteroids such as hydroxypregnene are decreased in the lactating stage but increased in pregnancy, which could be an indication of prescription of corticosteroids to treat symptoms of autoimmune conditions or of inflammation, as well as being one of the most important antenatal therapies available to improve newborn outcomes before anticipated preterm birth (El-Sayed et al., 2017). Also, interestingly, glucocorticoid compounds with anti-inflammatory and immunosuppressive effects are commonly used to treat inflammatory bowel disease, asthma, allergies, and rheumatic diseases and are upregulated among the pregnant group as well, denoting a normal behavior in pregnancy where maternal glucocorticoids critically rise reaching up to a 20-fold increase of mid-pregnancy concentrations (Solano and Arck, 2020). Also, in lactating women, we found an increased ratio of palmitoyl dopamine, which is an endogenous, long-chain, linear fatty acid dopamide with entourage effects in the endocannabinoid system (Matsumoto et al., 2016). This is of high interest since its biological significance in lactation is understudied and it is then an interesting metabolite to further analyze as a biomarker.

Continuing with deeper insights into the unique metabolic traits of each group of the cohort, it can be observed in Figures 3A,C, that sphingolipids and ceramides are upregulated among the lactating group. These metabolites are involved in the regulation of insulin resistance during the perinatal period (Rico et al., 2017). It is also abundant in human breast milk and has a positive impact on cognitive functions and brain development of the infant (Dei Cas et al., 2020). In addition, prostaglandins are known to affect uterine contractility and cervical ripening and are important in the initiation of labor (Wood et al., 2021). These we found as being upregulated in pregnant women, which denotes the correct reflection of the gut chemical environment with the state of the individual. Also, these findings are in accordance with what Liang and collaborators found in 2022. They found nine metabolites differentially expressed in stool samples from pregnant women in the third trimester and full term. These included levels of lipids and lipid-like molecules, such as long-chain fatty acids and 21-hydroxysteroids, being upregulated in pregnant women compared to full-term, whereas the levels of amino acids and dipeptides showed a downregulation. On the other hand, 20-hydroxyarachidonic acid and palmitic acid were enriched at the time of full-term pregnancy. Other metabolites like cyclohexylsulfamate, 3,3-dimethylacrylic acid, hydroxyisocaproic acid, and phenylalanylphenylalanine (Phe-Phe) were also identified in fecal samples from Chinese pregnant women (Liang et al., 2022).

In summary, for the chemical space composition of the gut or as called by us in this research, the gut metabolome of our cohort, we mainly observe metabolites that are either produced by the gut microbiome bacterial metabolism or modified by it. Examples of these compounds are bile acids, bilirubin (van Best et al., 2020; Garcia et al., 2022), tryptophan (Stoll et al., 2016; Gao et al., 2020), hormones (Jiang et al., 2021; Marć et al., 2022), glycerophosphocholines. Thus, we can suggest that the gut metabolome can be seen as a reflection of an appropriate gut microbiome profile, understating appropriate as the balance of the communities according to what has been reported for a healthy state in a certain condition. Also, we prove that fecal samples, which contain small and large molecules from the gut microbiome, can indeed reflect the net result of nutrient ingestion, digestion, and absorption by both gut bacteria and the gastrointestinal tract (Ulaszewska et al., 2019.)

Another interesting finding in this study, regarding a more general behavior of the population, is the reinforcement that gut metabolomics reflects diet, drug consumption, and pharmacokinetics, even if the person does not declare it or the initial data collected in the enrolling questionnaires, such as the one used in this study. For example, metabolites such as coumarin, omega-3 and omega-6 fatty acids, and vitamin E were detected, which are associated mostly with a plant-based diet (Pistollato et al., 2015; Sebastiani et al., 2019); drugs such as antihistamines and anticoagulants were found in the volunteers that declared its consumption. Central nervous system stimulants like caffeine were found significantly higher in the control group, and it is coherent with behavior during pregnancy and lactation, a time when women avoid high doses of this metabolite.

These findings are in accordance with the ones by (Pires et al., 2019), where the authors found significant metabolic changes in the chemical ecology of the gut environment between populations of individuals living in the Amazon, and those from an urban, industrialized setting, which was mainly attributed to dietary differences as well as diverse patterns of environmental exposure. Furthermore, organosilicons and other toxins coming from the heating of food, plastics, and agrochemicals, which can be harmful as they accumulate over time only when they have small particle sizes, are significantly abundant among the group of pregnant women from our region (Antioquia) which is not an encouraging finding from the public health perspective. Considering these compounds, specifically, those with a low silicon particle size, can overcome biological membranes and skin barriers, being possibly transferred to the baby (Dixon and Williamson, 2016), and can be endocrine disruptors. Organosilicon compounds are widely encountered in commercial products such as sealants, adhesives, coatings, medical products, and cosmetics (Mojsiewicz-Pienkowska et al., 2016).

Additionally, other, natural, compounds were found at toxic levels suggesting the ability of the methodology followed in the study to detect abnormal levels of naturally present molecules. Such is the case of volunteer 14, part of the pregnant women group, who showed significantly higher levels of primary bile acids which could be related to cholestasis and cause irreversible toxicity to the fetus (Mazzella et al., 2001).

We would like to highlight as well, that studies in other populations different from the American and European ones, such as Latin peoples, Asian people, African people, or women and children, are urgently needed as well as obataining the underlying data to properly acknowledge the gut microbiome and its associated chemical space on a world-populationscale. This will allow an appropriate and significant characterization of the gut microbiome of other countries and regions, as well as of different conditions such as healthy pregnancies and lactation in women. Furthermore, gut microbial communities change with age and sex; with one study showing a strong positive association between age and alpha diversity in young adults (less than 40 years old), and women were found to have more diversity than men (De la Cuesta-Zuluaga et al., 2019), thus groups like women, which have been previously excluded from study cohort in microbiome studies, should be included. Finally, there is a need to convert findings like the ones in this study into affordable and accessible strategies to measure gut health in every population. This reinforces the need for more studies on the gut metabolome in larger, and the use of ordinated (e.g., PCAs), clustering approaches, supervised models, or the employment of unsupervised models like NMF which have the added advantage that pre-calculated signatures of bacterial assemblages can be reapplied to even a single metagenome, removing the need for large cohort sizes capturing microbiome variation (Frioux et al., 2023).

Overall, this exploratory study serves as a starting point to describe the gut metabolome of healthy pregnant and lactating women from Antioquia, Colombia, a special population regarding the requirements of these physiological states and the profound impact that maternity can have on child development in terms of healthy growth, but also in its adequate cognitive development, as well as its regional nature. The two approaches to analyzing metabolomics data were complementary in the study, we could say that molecular networking serves as the starting point to have a broad panoramic view of the metabolites present in the chemical space. These can be later found in a more stringent and quantitative way by the untargeted metabolomic analysis.

## Conclusion

Gut metabolomics studies can shed light on the phenotype differences of a population with a specific condition, such as healthy pregnancies and lactation, from others. In this study, within a cohort of women from Antioquia, Colombia, we found that lactating women can be differentiated from other pregnant and reproductive-age non-pregnant nor lactating women by a gut metabolomic profile enriched in carnitine derivatives, glycerophosphocholines, bile acids, ceramides, glycerolipids, and glycerophosphoserines. Pregnant and lactating women, when compared to reproductive age controls, are enriched in glycerophosphocholines and glycerophosphoserines, corticosteroid hormones, bile acids, fatty acids, carbohydrates, and sterols. These metabolites can be further studied in a larger population, to scale their occurrence, and plausibly develop preventive biomarkers for healthy pregnancies. Metabolites such as toxins, xenobiotics, and environmental contaminants, which can be missed by other techniques, and are ubiquitous harmful foreign chemicals present in the environment, were detected in this study in fertile age, non-pregnant nor lactating women denoting a presence in the diet and lifestyle of women that can become pregnant in the future, posing a risk to the infant’s health. The metabolite Palmitoyl domamine was found as upregulated in lactating women, being reported for the first time in a gut metabolomics study, and in this specific population. Fibers and phytonutrients such as lignans and neolignans, glycosides, and lipid-derivatives from plant food sources, plant polyphenols, raffinose trisaccharides, glucosinolates, and lipopolysaccharides which are proven prebiotic compounds, were also found by molecular networking analysis in this cohort, denoting the capacity of this method to detect dietary compounds. Although our cohort is still limited for scaling these conclusions to a population level, this research sets an initial basis in our country and region, Latin America, for future population level measurements of a normal gut metabolome composition during the important perinatal period, which can provide valuable information to enhance public health nutrition strategies in middle-income countries.

## Data Availability

The datasets presented in this study can be found in online repositories. The names of the repository/repositories and accession number(s) can be found in the article/Supplementary Material.
