# Supplementary material for: The gut metabolome in a cohort of pregnant and lactating women from Antioquia-Colombia
Source: Front Mol Biosci. 2024 May 13;11:1250413. doi: 10.3389/fmolb.2024.1250413 (PMC11128665; doi:10.3389/fmolb.2024.1250413)
Supplement: Supplementary file 2 [file DataSheet1.PDF]

Metabolomic ID	State	Volunteer	Total Cholesterol	LDL	HDL	Triglycerides	BMI	Age	Height	Weight	gestational week	Postpartum week
**G1_001**	Lactating	1	335.3	239.46	56	199.2	24.386	34	1.62	64	0	0
**G1_002**	Lactating	2	153.7	76.86	65.9	54.7	25	35	1.6	64	20	20
**G1_003**	Lactating	3	213.4	138.76	57.9	83.7	19.487	32	1.68	55	4	4
**G1_004**	Lactating	4	203.3	128.78	50.1	122.1	N/D	N/D	N/D	N/D	0	0
**G2_010**	Pregnant	5	262.4	145.62	93.6	115.9	26.292	31	1.62	69	28	0
**G1_005**	Lactating	6	263.4	170.86	63.2	146.7	21.231	35	1.58	53	0	1
**G2_011**	Pregnant	7	321.3	194.78	75.7	254.1	18.671	32	1.62	49	32	0
**G2_012**	Pregnant	8	217.5	107.4	65.5	223	24.287	23	1.59	61.4	31	0
**G2_013**	Pregnant	9	213.1	106.1	71.9	175.5	29.752	35	1.65	81	30	0
**G2_014**	Pregnant	10	245.4	114.06	102.8	142.7	19.814	27	1.62	52	24	0
**G1_006**	Lactating	11	214.7	122.66	73.6	92.2	19.628	30	1.58	49	0	14
**G1_007**	Lactating	12	147.8	74	66.9	34.5	23.233	30	1.58	58	0	50
**G2_015**	Pregnant	13	159.6	70	56.6	165	38.935	23	1.65	106	25	0
**G2_016**	Pregnant	14	281.7	157	86.4	190.6	22.1	29	1.62	58	21	0
**G3_017**	Control	15	214.4	130.2	65.9	91.5	20.727	34	1.68	58.5	0	0
**G3_018**	Control	16	169.5	101.64	52.3	77.8	20.16	23	1.68	56.9	0	0
**G3_019**	Control	17	107.9	49.32	47.7	54.4	21.83	28	1.63	58	0	0
**G3_020**	Control	18	164.6	72.48	83.3	44.1	20.701	23	1.63	55	0	0
**G3_021**	Control	19	186.6	108.62	62.4	77.9	20.324	27	1.63	54	0	0
**G3_022**	Control	20	194.8	130.8	47.9	80.5	23.225	54	1.66	64	0	0
**G3_023**	Control	21	191.7	117.3	63.9	51	22.667	24	1.5	51	0	0
**G1_008**	Lactating	22	217.6	139.4	66.4	59.3	24.221	33	1.7	70	0	24
**G1_009**	Lactating	23	190.9	75.5	60.6	115.2	22.959	34	1.63	61	0	7
