# Supplementary material for: The gut metabolome in a cohort of pregnant and lactating women from Antioquia-Colombia
Source: Front Mol Biosci. 2024 May 13;11:1250413. doi: 10.3389/fmolb.2024.1250413 (PMC11128665; doi:10.3389/fmolb.2024.1250413)
Supplement: Supplementary file 3 [file Table3.DOCX]

|  |  |  |  |  |  |  |  |  |  | **Lactanting vs Control** | | |
| --- | --- | --- | --- | --- | --- | --- | --- | --- | --- | --- | --- | --- |
| **Compound** | **Formula** | **Mass** | **RT (min)** | **Mass Error (ppm)** | **Adduct** | **^a^CV for QC (%)** | **Analytical platform** | **DET** | **ID Level** | **^b^Fold Change** | **^c^VIP** | ***^d^p* value** |
| ***Amines*** |  |  |  |  |  |  |  |  |  |  |  |  |
| Hydroxyphenethylamine | C_8_H_11_NO | 137.0841 | 1.42 | 2 | [M+H-H_2_O]^+^ | 4.03 | RP-LC/MS | ESI + | 2 | 0.5 | 1.34 | 1.64E-02 |
| ***Amino acids and derivatives*** |  |  |  |  |  |  |  |  |  |  |  |  |
| Oleoyl phenylalanine | C_27_H_43_NO_3_ | 429.3243 | 7.4 | 3 | [M+H-H_2_O]^+^ | 3.66 | RP-LC/MS | ESI + | 3 | 7.6 | 1.55 | 2.29E-02 |
| Docosanoyl taurine | C_24_H_49_NO_4_S | 447.3382 | 8.7 | 3 | [M+H-H_2_O]^+^ | 4.69 | RP-LC/MS | ESI + | 3 | 2.8 | 1.87 | 4.18E-02 |
| Arachidonoyl glutamic acid | C_25_H_39_NO_5_ | 433.2828 | 6.55 | 1 | [M+K]^+^ | 2.60 | RP-LC/MS | ESI + | 3 | 0.4 | 2.39 | 4.18E-02 |
| Glutamylamino butanoate | C_9_H_16_N_2_O_5_ | 232.1059 | 7.02 | 1 | [M-H]^-^ | 0.91 | HILIC-LC/MS | ESI - | 3 | 0.2 | 1.87 | 1.15E-02 |
| Acetylglutamate | C_7_H_11_NO_5_ | 189.0637 | 5.81 | 2 | [M-H]^-^ | 0.81 | HILIC-LC/MS | ESI - | 2 | 0.5 | 1.51 | 3.11E-02 |
| ***Aldehydes*** |  |  |  |  |  |  |  |  |  |  |  |  |
| Phenylacetaldehyde | C_8_H_8_O | 120.0575 | 0.92 | 6 | [M+H]^+^ | 9.49 | RP-LC/MS | ESI + | 2 | 0.2 | 1.40 | - |
| ***Alkaloids*** |  |  |  |  |  |  |  |  |  |  |  |  |
| Methyl-tetradecanoyl-capnine | C_32_H_65_NO_5_S | 575.4583 | 0.54 | 4 | [M-H]^-^ | 3.31 | HILIC-LC/MS | ESI - | 3 | 1.9 | 1.78 | 2.29E-02 |
| Methyl-butanoyloxy-villanovane-diol | C_25_H_42_O_6_ | 438.2981 | 0.79 | 2 | [M-H]^-^ | 2.15 | HILIC-LC/MS | ESI - | 3 | 1.9 | 1.93 | 2.29E-02 |
| ***Benzoic acids*** |  |  |  |  |  |  |  |  |  |  |  |  |
| Malonyl aminobenzoic acid | C_10_H_9_NO_5_ | 223.0481 | 3.11 | 1 | [M-H]^-^ | 1.56 | HILIC-LC/MS | ESI - | 3 | 4.2 | 1.33 | 4.18E-02 |
| Phthalic acid | C_8_H_6_O_4_ | 166.0266 | 9.27 | 4 | [M+H-H_2_O]^+^ | 2.58 | RP-LC/MS | ESI + | 2 | 0.4 | 2.21 | 2.10E-03 |
| Phenylethyl acetate | C_10_H_12_O_2_ | 164.0837 | 13.98 | 5 | [M+H]^+^ | 1.64 | RP-LC/MS | ESI + | 2 | 0.4 | 1.67 | - |
| ***Bilirubins and biliverdins**** |  |  |  |  |  |  |  |  |  |  |  |  |
| Urobilinogen | C_33_H_48_N_4_O_6_ | 596.3574 | 9.58 | 0 | [M+K]^+^ | 18.09 | RP-LC/MS | ESI + | 3 | 0.2 | 2.04 | 1.64E-02 |
| ***Bile acids*** |  |  |  |  |  |  |  |  |  |  |  |  |
| Taurohyocholate | C_26_H_45_NO_7_S | 515.2917 | 2.26 | 0 | [M-H]^-^ | 0.49 | HILIC-LC/MS | ESI - | 3 | 4.0 | 2.06 | 7.87E-03 |
| Hydroxy-3-oxo-5-cholan-24-oic Acid | C_24_H_38_O_4_ | 390.277 | 9.48 | 4 | [M+H]^+^ | 2.24 | RP-LC/MS | ESI + | 3 | 3.2 | 1.36 | 1.64E-02 |
| Dihydroxy-5-cholan-24-oic Acid | C_24_H_40_O_4_ | 392.2927 | 11.02 | 4 | [M+Na]^+^ | 4.45 | RP-LC/MS | ESI + | 3 | 1.4 | 2.36 | 6.99E-04 |
| [ST 24:1;O5](https://www.lipidmaps.org/resources/tools/chemdb_ontology?abbrev=ST%2024%3A1%3BO5) | C_24_H_40_O_5_ | 408.2876 | 0.96 | 0 | [M-H]^-^ | 1.79 | HILIC-LC/MS | ESI - | 3 | 1.7 | 1.06 | 3.11E-02 |
| [ST 24:2;O4](https://www.lipidmaps.org/resources/tools/chemdb_ontology?abbrev=ST%2024%3A2%3BO4) | C_24_H_38_O_4_ | 390.277 | 0.73 | 0 | [M-H]^-^ | 3.90 | HILIC-LC/MS | ESI - | 3 | 3.2 | 1.13 | 1.15E-02 |
| ST 24:1;O4 | C_24_H_40_O_4_ | 392.2927 | 0.75 | 6 | [M-H]^-^ | 2.85 | HILIC-LC/MS | ESI - | 3 | 2.0 | 2.33 | 7.87E-03 |
| 5-Cholanoic acid | C_24_H_40_O_2_ | 360.3028 | 9.05 | 6 | [M+H-H_2_O]^+^ | 1.73 | RP-LC/MS | ESI + | 3 | 4.6 | 1.81 | 4.18E-02 |
| ***Carbohydrates*** |  |  |  |  |  |  |  |  |  |  |  |  |
| Acetyllactosamine | C_14_H_25_NO_11_ | 383.1428 | 5.55 | 1 | [M+Cl]^-^ | 2.67 | HILIC-LC/MS | ESI - | 3 | 2.9 | 1.93 | 3.11E-02 |
| Acetylglucosamine | C_8_H_15_NO_6_ | 221.0899 | 4.55 | 2 | [M-H-H_2_O]^-^ | 5.49 | HILIC-LC/MS | ESI - | 3 | 0.1 | 1.71 | 2.10E-03 |
| ***Carbonyl compounds*** |  |  |  |  |  |  |  |  |  |  |  |  |
| Acetylthiazole | C_5_H_5_NOS | 127.0092 | 1.34 | 3 | [M-H]^-^ | 0.27 | HILIC-LC/MS | ESI - | 3 | 2.5 | 2.14 | 7.87E-03 |
| ***Carnitines**** |  |  |  |  |  |  |  |  |  |  |  |  |
| CAR 12:0 | C_19_H_37_NO_4_ | 343.2723 | 0.9 | 2 | [M-H]^-^ | 1.86 | HILIC-LC/MS | ESI - | 3 | 2.8 | 1.52 | 1.15E-02 |
| Palmitoylcarnitine | C_23_H_45_NO_4_ | 399.3349 | 13.07 | 5 | [M+H]^+^ | 2.26 | RP-LC/MS | ESI + | 2 | 2.6 | 1.04 | - |
| ***Ceramides*** |  |  |  |  |  |  |  |  |  |  |  |  |
| PI-Cer(d40:1) | C_46_H_90_NO_11_P | 863.6252 | 13.83 | 6 | [M+H]^+^ | 13.91 | RP-LC/MS | ESI + | 3 | 4.2 | 2.03 | 2.29E-02 |
| C17 Sphingosine | C_17_H_35_NO_2_ | 285.2668 | 10.5 | 5 | [M+H-H_2_O]^+^ | 4.62 | RP-LC/MS | ESI + | 3 | 4.0 | 1.72 | 3.11E-02 |
| Erythro-Sphingosine C-15 | C_15_H_31_NO_2_ | 257.2355 | 9.05 | 5 | [M+H-H_2_O]^+^ | 1.53 | RP-LC/MS | ESI + | 3 | 3.3 | 1.86 | 4.18E-02 |
| ***Corticosteroid hormones*** |  |  |  |  |  |  |  |  |  |  |  |  |
| Pregnanetriol | C_21_H_36_O_3_ | 336.2664 | 15.56 | 4 | [M+H]^+^ | 5.77 | RP-LC/MS | ESI + | 3 | 3.4 | 2.22 | 1.15E-02 |
| Dinorergosta-4,22-dien-3-one | C_26_H_40_O | 368.3079 | 10.66 | 4 | [M+H]^+^ | 3.03 | RP-LC/MS | ESI + | 3 | 3.7 | 1.34 | 3.11E-02 |
| Hydroxypregnene | C_21_H_34_O | 302.261 | 11.21 | 3 | [M+H-H_2_O]^+^ | 13.47 | RP-LC/MS | ESI + | 3 | 0.3 | 2.24 | 1.64E-02 |
| ***Ecdysones*** |  |  |  |  |  |  |  |  |  |  |  |  |
| Trihydroxyecdysone | C_27_H_44_O_9_ | 512.2985 | 4.18 | 5 | [M+H-H_2_O]^+^ | 5.48 | RP-LC/MS | ESI + | 3 | 0.2 | 1.75 | 2.29E-02 |
| ***Fatty acids*** |  |  |  |  |  |  |  |  |  |  |  |  |
| Keto stearic acid | C_18_H_34_O_3_ | 298.2508 | 10.63 | 4 | [M+H]^+^ | 6.94 | RP-LC/MS | ESI + | 2 | 0.5 | 2.35 | 5.24E-03 |
| Oxo-nonadecanoic acid | C_19_H_36_O_3_ | 312.2664 | 11.71 | 5 | [M+H]^+^ | 11.38 | RP-LC/MS | ESI + | 3 | 0.5 | 1.54 | 3.11E-02 |
| Hydroxy-heptadecatrienoic acid | C_17_H_28_O_3_ | 280.2038 | 6.09 | 1 | [M+K]^+^ | 2.24 | RP-LC/MS | ESI + | 3 | 0.5 | 2.07 | 4.18E-02 |
| Propylmalic acid | C_7_H_12_O_5_ | 176.0685 | 4.55 | 3 | [M-H]^-^ | 6.74 | HILIC-LC/MS | ESI - | 3 | 0.3 | 1.42 | 2.29E-02 |
| Decatrienoic acid | C_10_H_14_O_2_ | 166.0994 | 14.48 | 3 | [M+H-H_2_O]^+^ | 4.17 | RP-LC/MS | ESI + | 2 | 0.3 | 2.17 | 2.29E-02 |
| Heptadecatrienoic acid | C_17_H_28_O_2_ | 264.2089 | 5.56 | 3 | [M+K]^+^ | 14.24 | RP-LC/MS | ESI + | 3 | 3.1 | 2.21 | 1.15E-02 |
| Methyl-octadecatrienoic acid | C_19_H_32_O_2_ | 292.2402 | 14.75 | 3 | [M+H-H_2_O]^+^ | 3.09 | RP-LC/MS | ESI + | 2 | 8.2 | 2.46 | 1.15E-02 |
| Phenyl-heptadecenoic acid | C_23_H_36_O_2_ | 344.2715 | 9.48 | 4 | [M+H-H_2_O]^+^ | 1.24 | RP-LC/MS | ESI + | 3 | 3.2 | 1.34 | 1.64E-02 |
| ***Fatty amides*** |  |  |  |  |  |  |  |  |  |  |  |  |
| Palmitoyl dopamine | C_24_H_41_NO_3_ | 391.3086 | 9.71 | 4 | [M+H-H_2_O]^+^ | 2.46 | RP-LC/MS | ESI + | 3 | 2.9 | 1.87 | 4.18E-02 |
| (2'-(4-benzenesulfonamide)-ethyl) arachidonoyl amine | C_28_H_42_N_2_O_3_S | 486.2916 | 13.41 | 3 | [M+H]^+^ | 12.18 | RP-LC/MS | ESI + | 3 | 0.6 | 2.00 | 3.11E-02 |
| Hydroxy-eicosanoyl-homoserine lactone | C_24_H_45_NO_4_ | 411.3349 | 14.16 | 5 | [M+H]^+^ | 9.77 | RP-LC/MS | ESI + | 4 | 0.2 | 1.97 | 2.29E-02 |
| Docosanamide | C_22_H_45_NO | 339.3501 | 9.96 | 2 | [M+K]^+^ | 18.61 | RP-LC/MS | ESI + | 3 | 0.5 | 1.99 | 2.29E-02 |
| ***Glycerolipids*** |  |  |  |  |  |  |  |  |  |  |  |  |
| MG(18:2) | C_21_H_38_O_4_ | 354.277 | 14.47 | 4 | [M+H-H_2_O]^+^ | 19.74 | RP-LC/MS | ESI + | 3 | 2.1 | 1.80 | 4.18E-02 |
| MG(18:3) | C_21_H_36_O_4_ | 352.2614 | 11.23 | 5 | [M+H]^+^ | 3.34 | RP-LC/MS | ESI + | 3 | 1.8 | 1.28 | 4.18E-02 |
| MGDG-O(16:3) | C_43_H_68_O_11_ | 760.4762 | 8.16 | 3 | [M+H]^+^ | 1.29 | RP-LC/MS | ESI + | 4 | 4.2 | 2.45 | 4.18E-02 |
| ***Glycerophosphoglycerols*** |  |  |  |  |  |  |  |  |  |  |  |  |
| PHODA-PG | C_34_H_63_O_12_P | 694.4057 | 9.62 | 5 | [M+Na]^+^ | 3.53 | RP-LC/MS | ESI + | 3 | 0.3 | 2.49 | 1.64E-02 |
| LPG O-18:0 | C_24_H_51_O_8_P | 498.3322 | 1.52 | 4 | [M-H]^-^ | 2.89 | HILIC-LC/MS | ESI - | 3 | 0.3 | 2.07 | 1.64E-02 |
| PG(40:6) | C_46_H_79_O_10_P | 822.5411 | 11.01 | 1 | [M+H]^+^ | 2.94 | RP-LC/MS | ESI + | 3 | 0.7 | - | 4.18E-02 |
| PG 42:9 | C_48_H_77_O_10_P | 844.5254 | 1.4 | 2 | [M-H]^-^ | 5.50 | HILIC-LC/MS | ESI - | 3 | 2.9 | 2.26 | 2.10E-03 |
| PG O-28:1//PG P-28:0 | C_34_H_67_O_9_P | 650.4523 | 0.84 | 4 | [M-H]^-^ | 0.65 | HILIC-LC/MS | ESI - | 3 | 3.0 | 1.60 | 1.15E-02 |
| ***Glycerophosphocholines*** |  |  |  |  |  |  |  |  |  |  |  |  |
| LPC(18:2) | C_26_H_50_NO_7_P | 519.3325 | 11.38 | 4 | [M+H]^+^ | 11.26 | RP-LC/MS | ESI + | 2 | 1.8 | 1.12 | - |
| ***Glycerophosphoethanolamines*** |  |  |  |  |  |  |  |  |  |  |  |  |
| PE(38:9) | C_43_H_68_NO_8_P | 757.4683 | 5.87 | 0 | [M+H-H_2_O]^+^ | 15.39 | RP-LC/MS | ESI + | 3 | 0.4 | 1.88 | 2.29E-02 |
| PE(37:7) | C_42_H_70_NO_8_P | 747.4839 | 5.3 | 3 | [M+Na]^+^ | 0.74 | RP-LC/MS | ESI + | 3 | 0.4 | 1.66 | 4.18E-02 |
| PE O-30:1//PE P-30:0 | C_35_H_70_NO_7_P | 647.489 | 0.92 | 0 | [M-H]^-^ | 13.11 | HILIC-LC/MS | ESI - | 3 | 2.8 | 2.19 | 3.32E-03 |
| ***Glycerophosphoinositols*** |  |  |  |  |  |  |  |  |  |  |  |  |
| [PI O-32:1// PI P-32:0](https://www.lipidmaps.org/resources/tools/chemdb_ontology?abbrev=PI%20O-32%3A1) | C_41_H_79_O_12_P | 794.5309 | 1.4 | 0 | [M+Cl]^-^ | 2.80 | HILIC-LC/MS | ESI - | 3 | 4.2 | 2.20 | 2.10E-03 |
| LPI(16:0) | C_25_H_49_O_12_P | 572.2962 | 3.53 | 2 | [M-H]^-^ | 1.21 | HILIC-LC/MS | ESI - | 2 | 0.3 | 1.23 | 2.29E-02 |
| ***Glycerophosphoserines*** |  |  |  |  |  |  |  |  |  |  |  |  |
| PS(26:0) | C_32_H_62_NO_10_P | 651.4111 | 5.65 | 10 | [M+H]^+^ | 2.77 | RP-LC/MS | ESI + | 3 | 2.5 | 1.61 | 1.64E-02 |
| ***Glycosides*** |  |  |  |  |  |  |  |  |  |  |  |  |
| Hydroxyproline galactoside | C_11_H_19_NO_8_ | 293.1111 | 4.55 | 0 | [M-H]^-^ | 8.54 | HILIC-LC/MS | ESI - | 3 | 0.6 | 1.10 | 4.18E-02 |
| ***Lignan lactones*** |  |  |  |  |  |  |  |  |  |  |  |  |
| Dehydrodiferulic dilactone | C_20_H_18_O_8_ | 386.1002 | 7.5 | 3 | [M-H]^-^ | 1.30 | HILIC-LC/MS | ESI - | 3 | 1.7 | 1.89 | 3.11E-02 |
| ***Nucleosides*** |  |  |  |  |  |  |  |  |  |  |  |  |
| Deoxyinosine | C_10_H_12_N_4_O_4_ | 252.0859 | 5.66 | 7 | [M-H]^-^ | 3.17 | HILIC-LC/MS | ESI - | 3 | 0.1 | 1.44 | 3.11E-02 |
| Dimethylguanosine | C_12_H_17_N_5_O_5_ | 311.123 | 5.47 | 3 | [M-H-H2O]^-^ | 3.22 | HILIC-LC/MS | ESI - | 3 | 0.5 | 1.59 | 2.29E-02 |
| ***Peptides and proteins*** |  |  |  |  |  |  |  |  |  |  |  |  |
| Asp Ile | C_10_H_18_N_2_O_5_ | 246.1216 | 6.46 | 1 | [M-H]^-^ | 2.17 | HILIC-LC/MS | ESI - | 3 | 0.2 | 2.13 | 6.99E-04 |
| Glutamylleucine | C_11_H_20_N_2_O_5_ | 260.1372 | 6.58 | 1 | [M-H]^-^ | 0.72 | HILIC-LC/MS | ESI - | 2 | 0.4 | 2.03 | 3.11E-02 |
| Glutamylalanine | C_8_H_14_N_2_O_5_ | 218.0903 | 7.72 | 2 | [M-H]^-^ | 1.45 | HILIC-LC/MS | ESI - | 3 | 0.2 | 2.28 | 2.10E-03 |
| S-Decyl GSH | C_20_H_37_N_3_O_6_S | 447.2403 | 6.92 | 0 | [M+Na]^+^ | 5.07 | RP-LC/MS | ESI + | 3 | 0.5 | 2.34 | 1.15E-02 |
| His Ala Gln | C_14_H_22_N_6_O_5_ | 354.1652 | 5.66 | 2 | [M-H-H_2_O]^-^ | 2.90 | HILIC-LC/MS | ESI - | 3 | 0.4 | 1.20 | 4.18E-02 |
| ***Prostaglandins*** |  |  |  |  |  |  |  |  |  |  |  |  |
| PGF2 acetate methyl ester | C_23_H_38_O_6_ | 410.2668 | 11.86 | 4 | [M+Na]^+^ | 14.81 | RP-LC/MS | ESI + | 3 | 0.5 | 1.92 | 2.29E-02 |
| ***Polyketides*** |  |  |  |  |  |  |  |  |  |  |  |  |
| Porosuphenol B | C_27_H_38_O_6_ | 458.2668 | 0.96 | 1 | [M-H-H2O]^-^ | 1.88 | HILIC-LC/MS | ESI - | 3 | 0.5 | 2.11 | 1.64E-02 |
| ***Steroids*** |  |  |  |  |  |  |  |  |  |  |  |  |
| Cholestane | C_27_H_48_ | 372.3756 | 14.88 | 2 | [M+Na]^+^ | 6.78 | RP-LC/MS | ESI + | 3 | 0.2 | 2.53 | 3.32E-03 |
| Epidioxy-cholestadienol | C_27_H_42_O_3_ | 414.3134 | 11.68 | 4 | [M+H]^+^ | 4.56 | RP-LC/MS | ESI + | 3 | 0.5 | 2.63 | 6.99E-04 |
| Acetoxy-dihydroxy-seco-cholestenone | C_29_H_48_O_5_ | 476.3502 | 12.82 | 4 | [M+H-H_2_O]^+^ | 11.79 | RP-LC/MS | ESI + | 3 | 0.5 | 2.10 | 2.29E-02 |
| Chenodeoxycholic Acid | C_24_H_40_O_4_ | 392.2927 | 11 | 0 | [M+K]^+^ | 11.66 | RP-LC/MS | ESI + | 3 | 0.7 | - | 4.18E-02 |
| Tetrahydroxycholanoic Acid | C_24_H_40_O_6_ | 424.2825 | 6.71 | 9 | [M+Na]^+^ | 16.35 | RP-LC/MS | ESI + | 3 | 0.6 | 2.08 | 1.15E-02 |
| Dehydrocholic acid | C_24_H_34_O_5_ | 402.2406 | 8.23 | 10 | [M+Na]^+^ | 9.25 | RP-LC/MS | ESI + | 3 | 0.6 | 2.00 | 1.64E-02 |
| Hydroxy-oxo-cholestenoate | C_27_H_42_O_4_ | 430.3083 | 12.39 | 1 | [M+H]^+^ | 5.67 | RP-LC/MS | ESI + | 3 | 0.1 | 1.86 | 1.64E-02 |
| ***Sterols*** |  |  |  |  |  |  |  |  |  |  |  |  |
| Oxo-cholestenone | C_27_H_42_O_2_ | 398.3185 | 13.5 | 4 | [M+H]^+^ | 5.93 | RP-LC/MS | ESI + | 3 | 5.7 | 1.95 | 3.32E-03 |
| Methylenecholesterol | C_28_H_46_O | 398.3549 | 12 | 5 | [M+H]^+^ | 7.30 | RP-LC/MS | ESI + | 3 | 6.6 | 1.73 | 4.18E-02 |
| Norcholestenone | C_26_H_42_O | 370.3236 | 10.5 | 6 | [M+H]^+^ | 4.50 | RP-LC/MS | ESI + | 3 | 4.7 | 1.69 | 3.11E-02 |
| Sterol glucoside | C_23_H_38_O_6_ | 410.2668 | 9.88 | 0 | [M+H]^+^ | 3.82 | RP-LC/MS | ESI + | 3 | 2.7 | 1.74 | 3.11E-02 |
| Dihydroxycholesten-27-oic acid | C_27_H_44_O_4_ | 432.324 | 13.06 | 4 | [M+H]^+^ | 3.20 | RP-LC/MS | ESI + | 3 | 0.4 | 1.82 | 4.18E-02 |
| ***Saccharides*** |  |  |  |  |  |  |  |  |  |  |  |  |
| Sucrose monopalmitate | C_28_H_54_O_13_ | 598.3564 | 6.09 | 2 | [M+K]^+^ | 1.90 | RP-LC/MS | ESI + | 3 | 0.5 | 1.99 | 3.11E-02 |
| ***Vitamin D*** |  |  |  |  |  |  |  |  |  |  |  |  |
| Acetoxy-hydroxyvitamin D3 | C_29_H_46_O_4_ | 458.3396 | 13.49 | 4 | [M+H]^+^ | 8.82 | RP-LC/MS | ESI + | 3 | 0.5 | 2.05 | 3.11E-02 |
| ^a^CV, coefficient of variation in the metabolites in the QC samples; ^b^Fold change in the abundance of the specified comparison calculated as (case/control); ^c^VIP, variable importance in projection; ^d^ p value < 0.05. RP: reversed-phase, HILIC: Hydrophilic interaction chromatography, LC: liquid chromatography, MS: mass spectrometry. | | | | | | | | | | | | |

|  |  |  |  |  |  |  |  |  |  | **Pregnant vs Lactanting** | | |
| --- | --- | --- | --- | --- | --- | --- | --- | --- | --- | --- | --- | --- |
| **Compound** | **Formula** | **Mass** | **RT (min)** | **Mass Error (ppm)** | **Adduct** | **^a^CV for QC (%)** | **Analytical platform** | **DET** | **ID Level** | **^b^Fold Change** | **^c^VIP** | ***^d^p* value** |
| ***Amino acids*** |  |  |  |  |  |  |  |  |  |  |  |  |
| Homophenylalanine | C_10_H_13_NO_2_ | 179.0946 | 6.94 | 5 | [M+H]^+^ | 8.08 | RP-LC/MS | ESI + | 3 | 0.7 | 1.27 | - |
| ***Alkaloids*** |  |  |  |  |  |  |  |  |  |  |  |  |
| Piperine* | C_17_H_19_NO_3_ | 285.1365 | 9.99 | 4 | [M+H]^+^ | 2.15 | RP-LC/MS | ESI + | 2 | 18.7 | 2.33 | - |
| Methyl-tetradecanoyl-capnine | C_32_H_65_NO_5_S | 575.4583 | 0.54 | 4 | [M-H]^-^ | 3.31 | HILIC-LC/MS | ESI - | 3 | 0.2 | 2.30 | 3.32E-03 |
| ***Benzenoids*** |  |  |  |  |  |  |  |  |  |  |  |  |
| Phenylacetamide | C_8_H_9_NO | 135.0684 | 5.47 | 5 | [M+H]^+^ | 3.61 | RP-LC/MS | ESI + | 3 | 1.5 | 1.24 | - |
| Phthalic acid | C_8_H_6_O_4_ | 166.0266 | 9.27 | 4 | [M+H-H_2_O]^+^ | 2.58 | RP-LC/MS | ESI + | 2 | 2.0 | 1.53 | - |
| ***Carbonyl compounds*** |  |  |  |  |  |  |  |  |  |  |  |  |
| Acetylthiazole | C_5_H_5_NOS | 127.0092 | 1.34 | 3 | [M-H]^-^ | 0.27 | HILIC-LC/MS | ESI - | 3 | 1.3 | 1.43 | 2.29E-02 |
| ***Carboxylic acids*** |  |  |  |  |  |  |  |  |  |  |  |  |
| Diphenolic acid | C_17_H_18_O_4_ | 286.1205 | 1.36 | 3 | [M-H]^-^ | 2.65 | HILIC-LC/MS | ESI - | 3 | 0.4 | 1.95 | 3.11E-02 |
| ***Corticosteroid**** |  |  |  |  |  |  |  |  |  |  |  |  |
| Hydroxypregnene | C_21_H_34_O | 302.261 | 11.21 | 3 | [M+H-H_2_O]^+^ | 13.47 | RP-LC/MS | ESI + | 3 | 17.9 | 2.64 | 1.64E-02 |
| ***Fatty Acyls*** |  |  |  |  |  |  |  |  |  |  |  |  |
| Phenyl-heptadecenoic acid | C_23_H_36_O_6_ | 344.2715 | 9.48 | 4 | [M+H-H_2_O]^+^ | 1.24 | RP-LC/MS | ESI + | 3 | 0.2 | 1.49 | 1.64E-02 |
| Hexacosanedioic acid | C_26_H_50_O_4_ | 426.3709 | 0.77 | 2 | [M-H]^-^ | 3.51 | HILIC-LC/MS | ESI - | 3 | 0.2 | 2.19 | 5.24E-03 |
| FAHFA 28:0;O | C_28_H_54_O_4_ | 454.4022 | 0.77 | 2 | [M-H]^-^ | 3.36 | HILIC-LC/MS | ESI - | 3 | 0.2 | 1.61 | 3.11E-02 |
| FAHFA 32:0;O | C_32_H_62_O_4_ | 510.4648 | 0.73 | 2 | [M-H]^-^ | 4.45 | HILIC-LC/MS | ESI - | 3 | 0.5 | 1.28 | 4.18E-02 |
| Adipic acid | C_6_H_10_O_4_ | 146.0579 | 1.45 | 5 | [M-H]^-^ | 0.78 | HILIC-LC/MS | ESI - | 3 | 2.2 | 1.80 | 7.87E-03 |
| Octadecanedioic acid | C_18_H_34_O_4_ | 314.2457 | 0.79 | 5 | [M-H]^-^ | 5.37 | HILIC-LC/MS | ESI - | 3 | 3.4 | 1.80 | 1.64E-02 |
| ***Glycerolipids*** |  |  |  |  |  |  |  |  |  |  |  |  |
| DG 33:4 | C_36_H_62_O_5_ | 574.4597 | 0.46 | 0 | [M-H-H_2_O]^-^ | 0.76 | HILIC-LC/MS | ESI - | 3 | 0.4 | 1.66 | 2.29E-02 |
| ***Glycosides*** |  |  |  |  |  |  |  |  |  |  |  |  |
| Sterol glucoside | C_23_H_38_O_6_ | 410.2668 | 9.88 | 0 | [M+H]^+^ | 3.82 | RP-LC/MS | ESI + | 3 | 0.2 | 2.10 | - |
| ***Glycerophosphoglycerols*** |  |  |  |  |  |  |  |  |  |  |  |  |
| LPG O-16:0 | C_22_H_47_O_8_P | 470.3009 | 1.6 | 1 | [M-H]^-^ | 1.49 | HILIC-LC/MS | ESI - | 3 | 2.7 | 1.68 | 3.11E-02 |
| LPG(12:0) | C_18_H_37_O_9_P | 428.2175 | 1.79 | 1 | [M-H]^-^ | 1.37 | HILIC-LC/MS | ESI - | 2 | 0.4 | 2.05 | 3.32E-03 |
| PG 32:0 | C_38_H_75_O_10_P | 722.5098 | 0.56 | 6 | [M-H]^-^ | 10.24 | HILIC-LC/MS | ESI - | 3 | 0.4 | 1.96 | 2.29E-02 |
| PG O-28:1//PG P-28:0 | C_34_H_67_O_9_P | 650.4523 | 0.84 | 4 | [M-H]^-^ | 0.65 | HILIC-LC/MS | ESI - | 3 | 0.2 | 1.76 | 6.99E-04 |
| ***Glycerophosphocholines*** |  |  |  |  |  |  |  |  |  |  |  |  |
| LPC(18:1) | C_26_H_52_NO_7_P | 521.3481 | 12.29 | 4 | [M+H]^+^ | 9.48 | RP-LC/MS | ESI + | 2 | 0.5 | 1.10 | - |
| LPC(18:2) | C_26_H_50_NO_7_P | 519.3325 | 11.38 | 4 | [M+H]^+^ | 11.26 | RP-LC/MS | ESI + | 2 | 0.4 | 1.30 | - |
| SM C16:1 | C_40_H_80_N_2_O_6_P | 715.5754 | 0.87 | 1 | [M-H]^-^ | 0.58 | HILIC-LC/MS | ESI - | 3 | 0.3 | 2.05 | 5.24E-03 |
| PC 40:9 | C_48_H_78_NO_8_P | 827.5465 | 1.42 | 5 | [M-H-H_2_O]^-^ | 6.53 | HILIC-LC/MS | ESI - | 3 | 0.3 | 1.37 | 2.29E-02 |
| ***Glycerophosphoethanolamines*** |  |  |  |  |  |  |  |  |  |  |  |  |
| PE(38:4)(15Ke)) | C_43_H_76_NO_9_P | 781.5258 | 14.76 | 8 | [M+Na]^+^ | 11.87 | RP-LC/MS | ESI + | 3 | 2.6 | 2.68 | 2.10E-03 |
| PE(37:7) | C_42_H_70_NO_8_P | 747.4839 | 5.3 | 3 | [M+Na]^+^ | 0.74 | RP-LC/MS | ESI + | 3 | 4.9 | 1.10 | - |
| PE O-30:1//PE P-30:0 | C_35_H_70_NO_7_P | 647.489 | 0.92 | 0 | [M-H]^-^ | 13.11 | HILIC-LC/MS | ESI - | 3 | 0.3 | 2.17 | 2.10E-03 |
| PE(30:0) | C_35_H_70_NO_8_P | 663.4839 | 0.96 | 0 | [M-H]^-^ | 1.19 | HILIC-LC/MS | ESI - | 3 | 0.5 | 1.59 | 3.11E-02 |
| [PE 29:0](https://www.lipidmaps.org/resources/tools/chemdb_ontology?abbrev=PE%2029%3A0) | C_34_H_68_NO_8_P | 649.4683 | 0.95 | 3 | [M-H]^-^ | 1.00 | HILIC-LC/MS | ESI - | 3 | 0.5 | 1.69 | 3.11E-02 |
| ***Glycerophosphoserines*** |  |  |  |  |  |  |  |  |  |  |  |  |
| PS(26:0) | C_32_H_62_NO_10_P | 651.4111 | 5.65 | 10 | [M+H]^+^ | 2.77 | RP-LC/MS | ESI + | 3 | 3.5 | 1.12 | - |
| ***Glycerophosphates*** |  |  |  |  |  |  |  |  |  |  |  |  |
| PA O-34:3//PA P-34:2 | C_37_H_69_O_7_P | 656.4781 | 0.47 | 3 | [M-H-H_2_O]^-^ | 0.54 | HILIC-LC/MS | ESI - | 3 | 10.8 | 2.25 | 6.99E-04 |
| PA O-36:3//PA P-36:2 | C_39_H_73_O_7_P | 684.5094 | 0.47 | 3 | [M-H-H_2_O]^-^ | 2.85 | HILIC-LC/MS | ESI - | 3 | 5.5 | 2.18 | 7.87E-03 |
| ***Indoles*** |  |  |  |  |  |  |  |  |  |  |  |  |
| Hexacosanoyltryptamine | C_36_H_62_N_2_O | 538.4862 | 0.53 | 2 | [M+Cl]^-^ | 2.29 | HILIC-LC/MS | ESI - | 3 | 0.4 | 2.61 | 3.50E-04 |
| ***Nucleosides*** |  |  |  |  |  |  |  |  |  |  |  |  |
| Deoxyinosine | C_10_H_12_N_4_O_4_ | 252.0859 | 5.66 | 7 | [M-H]^-^ | 3.17 | HILIC-LC/MS | ESI - | 3 | 4.9 | 1.40 | 5.24E-03 |
| ***Organooxygen compounds*** |  |  |  |  |  |  |  |  |  |  |  |  |
| Arbutin | C_12_H_16_O_7_ | 272.0896 | 3.75 | 1 | [M-H]^-^ | 3.78 | HILIC-LC/MS | ESI - | 3 | 0.4 | 2.11 | 1.15E-02 |
| Acetylamino-amino-methyluracil | C_7_H_10_N_4_O_3_ | 198.0753 | 1.67 | 5 | [M-H]^-^ | 0.60 | HILIC-LC/MS | ESI - | 2 | 0.5 | 1.60 | 3.11E-02 |
| ***Peptides*** |  |  |  |  |  |  |  |  |  |  |  |  |
| Valyl-Leucine | C_11_H_22_N_2_O_3_ | 230.163 | 3.6 | 1 | [M-H]^-^ | 0.55 | HILIC-LC/MS | ESI - | 3 | 0.3 | 1.64 | 3.11E-02 |
| Threoninyl-Valine | C_9_H_18_N_2_O_4_ | 218.1267 | 5.62 | 0 | [M-H]^-^ | 1.11 | HILIC-LC/MS | ESI - | 2 | 0.3 | 1.56 | 4.18E-02 |
| ***Prostaglandins*** |  |  |  |  |  |  |  |  |  |  |  |  |
| Prostaglandin A1-biotin | C_35_H_58_N_4_O_5_S | 646.4128 | 7.7 | 10 | [M+K]^+^ | 13.61 | RP-LC/MS | ESI + | 3 | 6.0 | 2.66 | 6.99E-04 |
| Acetoxy-PGF2-acetate | C_24_H_38_O_8_ | 454.2567 | 1.17 | 5 | [M-H-H_2_O]^-^ | 13.89 | HILIC-LC/MS | ESI - | 3 | 0.5 | 1.18 | 4.18E-02 |
| ***Steroids*** |  |  |  |  |  |  |  |  |  |  |  |  |
| Glycocholic acid | C_26_H_43_NO_6_ | 465.309 | 1.11 | 5 | [M-H-H_2_O]^-^ | 4.08 | HILIC-LC/MS | ESI - | 3 | 2.5 | 2.81 | 6.99E-04 |
| Cholestane | C_27_H_48_ | 372.3756 | 14.88 | 2 | [M+Na]^+^ | 6.78 | RP-LC/MS | ESI + | 3 | 2.8 | 1.31 | - |
| [ST 24:1;O5;S](https://www.lipidmaps.org/resources/tools/chemdb_ontology?abbrev=ST%2024%3A1%3BO5%3BS) | C_24_H_40_O_8_S | 488.2444 | 1.13 | 2 | [M-H]^-^ | 0.62 | HILIC-LC/MS | ESI - | 2 | 2.4 | 2.14 | 1.64E-02 |
| ST 27:1;O2;S | C_27_H_46_O_5_S | 482.3066 | 0.79 | 2 | [M-H]^-^ | 3.87 | HILIC-LC/MS | ESI - | 3 | 1.8 | 1.56 | 2.29E-02 |
| Hydroxy-oxo-cholestenoate | C_27_H_42_O_4_ | 430.3083 | 12.39 | 1 | [M+H]^+^ | 5.67 | RP-LC/MS | ESI + | 3 | 1.5 | 1.03 | - |
| ***Sterol Lipids*** |  |  |  |  |  |  |  |  |  |  |  |  |
| Oxo-cholestenone | C_27_H_42_O_2_ | 398.3185 | 13.5 | 4 | [M+H]^+^ | 5.93 | RP-LC/MS | ESI + | 3 | 0.4 | 1.33 | - |
| Androstenol | C_19_H_30_O | 274.2297 | 14.75 | 3 | [M+H]^+^ | 3.09 | RP-LC/MS | ESI + | 2 | 0.4 | 1.62 | - |
| Hydroxy-oxocholanoic acid | C_24_H_38_O_4_ | 390.277 | 9.48 | 4 | [M+H]^+^ | 2.24 | RP-LC/MS | ESI + | 3 | 0.2 | 1.44 | 1.15E-02 |

^a^CV, coefficient of variation in the metabolites in the QC samples; ^b^Fold change in the abundance of the specified comparison calculated as (case/control); ^c^VIP, variable importance in projection; ^d^ p value < 0.05. RP: reversed-phase, HILIC: Hydrophilic interaction chromatography, LC: liquid chromatography, MS: mass spectrometry.

|  |  |  |  |  |  |  |  |  |  | **Pregnant vs Control** | | |
| --- | --- | --- | --- | --- | --- | --- | --- | --- | --- | --- | --- | --- |
| **Compound** | **Formula** | **Mass** | **RT (min)** | **Mass Error (ppm)** | **Adduct** | **^a^CV for QC (%)** | **Analytical platform** | **DET** | **ID Level** | **^b^Fold Change** | **^c^VIP** | ***^d^p* value** |
| ***Alkaloids*** |  |  |  |  |  |  |  |  |  |  |  |  |
| Piperine* | C_17_H_19_NO_3_ | 285.1365 | 9.99 | 4 | [M+H]^+^ | 2.15 | RP-LC/MS | ESI + | 2 | 2.4 | 1.16 | - |
| Tomatidanone | C_27_H_43_NO_2_ | 413.3294 | 7.97 | 3 | [M+H]^+^ | 1.17 | RP-LC/MS | ESI + | 3 | 3.4 | 1.23 | 3.79E-02 |
| Methyl-tetradecanoyl-capnine | C_32_H_65_NO_5_S | 575.4583 | 0.54 | 4 | [M-H]^-^ | 3.31 | HILIC-LC/MS | ESI - | 3 | 0.4 | 2.13 | 1.11E-02 |
| ***Amino acids and derivatives*** | | |  |  |  |  |  |  |  |  |  |  |
| Glutamylamino butanoate | C_9_H_16_N_2_O_5_ | 232.1059 | 7.02 | 1 | [M-H]^-^ | 0.91 | HILIC-LC/MS | ESI - | 3 | 0.2 | 1.59 | 2.62E-02 |
| ***Amines*** |  |  |  |  |  |  |  |  |  |  |  |  |
| Hydroxyphenethylamine | C_8_H_11_NO | 137.0841 | 1.42 | 2 | [M+H-H_2_O]^+^ | 4.03 | RP-LC/MS | ESI + | 2 | 0.6 | 1.05 | - |
| ***Benzenoids*** |  |  |  |  |  |  |  |  |  |  |  |  |
| Phenylacetaldehyde | C_8_H_8_O | 120.0575 | 0.92 | 6 | [M+H]^+^ | 9.49 | RP-LC/MS | ESI + | 2 | 0.1 | 1.20 | - |
| Dimethylaniline | C_8_H_11_N | 121.0891 | 7.27 | 4 | [M+H]^+^ | 1.88 | RP-LC/MS | ESI + | 2 | 0.4 | 1.06 | - |
| ***Bile acids*** |  |  |  |  |  |  |  |  |  |  |  |  |
| Cholanoic acid // Ursocholanic Acid | C_24_H_40_O_2_ | 360.3028 | 9.05 | 6 | [M+H-H_2_O]^+^ | 1.73 | RP-LC/MS | ESI + | 3 | 3.9 | 1.80 | - |
| Taurohyocholate | C_26_H_45_NO_7_S | 515.2917 | 2.26 | 0 | [M-H]^-^ | 0.49 | HILIC-LC/MS | ESI - | 3 | 3.1 | 1.63 | 1.75E-02 |
| Glycocholic acid | C_26_H_43_NO_6_ | 465.309 | 1.11 | 5 | [M-H-H_2_O]^-^ | 4.08 | HILIC-LC/MS | ESI - | 3 | 2.6 | 2.76 | 5.83E-04 |
| ***Bilirubins and biliverdins*** | |  |  |  |  |  |  |  |  |  |  |  |
| Urobilinogen | C_33_H_48_N_4_O_6_ | 596.3574 | 9.58 | 0 | [M+K]^+^ | 18.09 | RP-LC/MS | ESI + | 3 | 0.4 | 1.15 | - |
| ***Carbohydrates*** | |  |  |  |  |  |  |  |  |  |  |  |
| Methylthioribose | C_6_H_12_O_4_S | 180.0456 | 8.74 | 2 | [M+Na]^+^ | 4.09 | RP-LC/MS | ESI + | 3 | 2.6 | 1.80 | 2.62E-02 |
| ***Corticosteroid**** | |  |  |  |  |  |  |  |  |  |  |  |
| Hydroxypregnene | C_21_H_34_O | 302.261 | 11.21 | 3 | [M+H-H_2_O]^+^ | 13.47 | RP-LC/MS | ESI + | 3 | 5.4 | 1.94 | 2.62E-02 |
| Pregnanetriol | C_21_H_36_O_3_ | 336.2664 | 15.56 | 4 | [M+H]^+^ | 5.77 | RP-LC/MS | ESI + | 3 | 2.8 | 1.19 | - |
| ***Ecdysones*** |  |  |  |  |  |  |  |  |  |  |  |  |
| Trihydroxyecdysone | C_27_H_44_O_9_ | 512.2985 | 4.18 | 5 | [M+H-H_2_O]^+^ | 5.48 | RP-LC/MS | ESI + | 3 | 0.2 | 1.34 | - |
| ***Fatty Acyls*** |  |  |  |  |  |  |  |  |  |  |  |  |
| Heptadecatrienoic acid | C_17_H_28_NO_2_ | 264.2089 | 5.56 | 3 | [M+K]^+^ | 14.24 | RP-LC/MS | ESI + | 2 | 2.4 | 1.53 | - |
| Oleoyl phenylalanine | C_27_H_43_NO_3_ | 429.3243 | 7.4 | 3 | [M+H-H_2_O]^+^ | 3.66 | RP-LC/MS | ESI + | 3 | 8.2 | 2.04 | 5.83E-04 |
| Stearoylcarnitine | C_25_H_49_NO_4_ | 427.3662 | 11.69 | 8 | [M+Na]^+^ | 2.92 | RP-LC/MS | ESI + | 3 | 7.4 | 2.21 | 1.75E-02 |
| Docosanoyl taurine | C_24_H_49_NO_4_S | 447.3382 | 8.7 | 3 | [M+H-H_2_O]^+^ | 4.69 | RP-LC/MS | ESI + | 3 | 2.4 | 1.17 |  |
| Methyl-eicosapentaenoate | C_21_H_32_O_2_ | 316.2402 | 13.16 | 4 | [M+H]^+^ | 3.62 | RP-LC/MS | ESI + | 3 | 8.4 | - | 1.11E-02 |
| Hexacosatetraenoic acid | C_26_H_44_O_2_ | 388.3341 | 10.5 | 6 | [M+H-H_2_O]^+^ | 4.50 | RP-LC/MS | ESI + | 3 | 4.8 | 1.82 |  |
| Hydroxy stearic acid | C_18_H_36_O_3_ | 300.2664 | 13.62 | 5 | [M+H]^+^ | 6.54 | RP-LC/MS | ESI + | 3 | 0.3 | 1.74 | 2.62E-02 |
| Keto stearic acid | C_18_H_34_O_3_ | 298.2508 | 10.63 | 4 | [M+H]^+^ | 6.94 | RP-LC/MS | ESI + | 2 | 0.3 | 2.02 | 5.83E-04 |
| Decatrienoic acid | C_10_H_14_O_2_ | 166.0994 | 14.48 | 3 | [M+H-H_2_O]^+^ | 4.17 | RP-LC/MS | ESI + | 2 | 0.4 | 1.28 | - |
| Hydroxy-eicosanoyl-homoserine lactone | C_24_H_45_NO_4_ | 411.3349 | 14.16 | 5 | [M+H]^+^ | 9.77 | RP-LC/MS | ESI + | 4 | 0.2 | 1.56 | 2.62E-02 |
| Trimethyltridecatetraene | C_16_H_28_ | 220.2191 | 10.67 | 4 | [M+K]^+^ | 3.50 | RP-LC/MS | ESI + | 3 | 0.2 | 1.60 | 3.79E-02 |
| Docosahexaenoyl histidine | C_28_H_39_N_3_O_3_ | 465.2991 | 6.39 | 1 | [M+Na]^+^ | 5.34 | RP-LC/MS | ESI + | 3 | 0.3 | 1.79 | 2.62E-02 |
| Oleic acid | C_18_H_34_O_2_ | 282.2559 | 14.2 | 4 | [M+H-H_2_O]^+^ | 6.17 | RP-LC/MS | ESI + | 3 | 0.3 | 2.11 | 2.33E-03 |
| FAHFA 32:0;O | C_32_H_62_O_4_ | 510.4648 | 0.73 | 2 | [M-H]^-^ | 4.45 | HILIC-LC/MS | ESI - | 3 | 0.4 | 1.77 | 3.79E-02 |
| Octadecanedioic acid | C_18_H_34_O_4_ | 314.2457 | 0.79 | 5 | [M-H]^-^ | 5.37 | HILIC-LC/MS | ESI - | 3 | 6.0 | 1.99 | 5.83E-04 |
| ***Glycoside*** |  |  |  |  |  |  |  |  |  |  |  |  |
| Lipomycin | C_32_H_45_NO_9_ | 587.3094 | 7.34 | 9 | [M+H-H_2_O]^+^ | 2.27 | RP-LC/MS | ESI + | 3 | 0.3 | 1.75 | 2.62E-02 |
| Rhamnosyl-hydroxydecanoyl-hydroxydecanoicacid | C_27_H_50_O_9_ | 518.3455 | 4.16 | 9 | [M+Na]^+^ | 3.09 | RP-LC/MS | ESI + | 3 | 0.2 | 1.32 | 1.75E-02 |
| ***Glycerophosphoglycerols*** | |  |  |  |  |  |  |  |  |  |  |  |
| LPG O-18:0 | C_24_H_51_O_8_P | 498.3322 | 1.52 | 4 | [M-H]^-^ | 2.89 | HILIC-LC/MS | ESI - | 3 | 0.3 | 1.87 | 3.79E-02 |
| PHODA-PG | C_34_H_63_O_12_P | 694.4057 | 9.62 | 5 | [M+Na]^+^ | 3.53 | RP-LC/MS | ESI + | 3 | 0.3 | 2.53 | 2.33E-03 |
| PHODA-PG | C_34_H_63_O_12_P | 694.4057 | 9.76 | 6 | [M+Na]^+^ | 2.16 | RP-LC/MS | ESI + | 3 | 0.4 | 2.47 | 1.17E-03 |
| PS-PG | C_26_H_49_O_12_P | 584.2962 | 14.35 | 10 | [M+H-H_2_O]^+^ | 2.88 | RP-LC/MS | ESI + | 3 | 0.2 | 1.34 | 3.79E-02 |
| ***Glycerophosphocholines*** | |  |  |  |  |  |  |  |  |  |  |  |
| PC(30:2) | C_38_H_72_NO_8_P | 701.4996 | 5.96 | 4 | [M+K]^+^ | 6.17 | RP-LC/MS | ESI + | 3 | 13.0 | - | 4.08E-03 |
| OS-PC | C_30_H_56_NO_10_P | 621.3642 | 5.21 | 9 | [M+H]^+^ | 1.53 | RP-LC/MS | ESI + | 3 | 5.5 | - | 2.62E-02 |
| ***Glycerophosphoethanolamines*** | | |  |  |  |  |  |  |  |  |  |  |
| PE(38:9) | C_43_H_68_NO_8_P | 757.4683 | 5.87 | 0 | [M+H-H_2_O]^+^ | 15.39 | RP-LC/MS | ESI + | 3 | 0.1 | 2.38 | 4.08E-03 |
| PON-PE | C_30_H_58_NO_9_P | 607.3849 | 5.14 | 9 | [M+H]^+^ | 1.20 | RP-LC/MS | ESI + | 3 | 2.7 | 1.64 | 3.79E-02 |
| ***Glycerophosphoserines*** | |  |  |  |  |  |  |  |  |  |  |  |
| LPS(26:0) | C_32_H_62_NO_10_P | 651.4111 | 5.65 | 10 | [M+H]^+^ | 2.77 | RP-LC/MS | ESI + | 3 | 8.7 | - | 3.79E-02 |
| LPS(26:0) | C_32_H_62_NO_10_P | 651.4111 | 5.28 | 8 | [M+H]^+^ | 0.92 | RP-LC/MS | ESI + | 3 | 3.2 | 1.60 | 3.79E-02 |
| PS(34:6) | C_40_H_66_NO_10_P | 751.4424 | 12.22 | 8 | [M+H]^+^ | 11.97 | RP-LC/MS | ESI + | 3 | 3.1 | 1.91 | 2.62E-02 |
| PS(36:1) | C_42_H_80_NO_10_P | 789.552 | 5.76 | 3 | [M+K]^+^ | 2.14 | RP-LC/MS | ESI + | 3 | 8.3 | - | 3.79E-02 |
| PS(41:6) | C_47_H_80_NO_10_P | 849.552 | 5.86 | 4 | [M+Na]^+^ | 1.68 | RP-LC/MS | ESI + | 3 | 12.1 | - | 1.11E-02 |
| PS(39:6) | C_45_H_76_NO_10_P | 821.5207 | 14.76 | 4 | [M+H-H_2_O]^+^ | 11.87 | RP-LC/MS | ESI + | 3 | 3.3 | 2.22 | 4.08E-03 |
| ***Glycerophosphates*** | |  |  |  |  |  |  |  |  |  |  |  |
| PA O-34:3//PA P-34:2 | C_37_H_69_O_7_P | 656.4781 | 0.47 | 3 | [M-H-H_2_O]^-^ | 0.54 | HILIC-LC/MS | ESI - | 3 | 9.3 | 2.16 | 2.33E-03 |
| PA O-36:3//PA P-36:2 | C_39_H_73_O_7_P | 684.5094 | 0.47 | 3 | [M-H-H_2_O]^-^ | 2.85 | HILIC-LC/MS | ESI - | 3 | 5.6 | 2.13 | 1.11E-02 |
| ***Indoles*** |  |  |  |  |  |  |  |  |  |  |  |  |
| Hexacosanoyltryptamine | C_36_H_62_N_2_O | 538.4862 | 0.53 | 2 | [M+Cl]^-^ | 2.29 | HILIC-LC/MS | ESI - | 3 | 0.5 | 2.32 | 4.08E-03 |
| ***Organooxygen compounds*** | |  |  |  |  |  |  |  |  |  |  |  |
| Acetylthiazole | C_5_H_5_NOS | 127.0092 | 1.34 | 3 | [M-H]^-^ | 0.27 | HILIC-LC/MS | ESI - | 3 | 3.4 | 2.47 | 4.08E-03 |
| Arbutin | C_12_H_16_O_7_ | 272.0896 | 3.75 | 1 | [M-H]^-^ | 3.78 | HILIC-LC/MS | ESI - | 3 | 0.3 | 1.86 | 2.62E-02 |
| ***Peptides and proteins*** | |  |  |  |  |  |  |  |  |  |  |  |
| Glutamylleucine | C_11_H_20_N_2_O_5_ | 260.1372 | 6.58 | 1 | [M-H]^-^ | 0.72 | HILIC-LC/MS | ESI - | 2 | 0.3 | 1.89 | 2.62E-02 |
| ***Polyketides*** |  |  |  |  |  |  |  |  |  |  |  |  |
| Diacetoxy-oxo-lanostatrienoic acid | C_34_H_48_O_7_ | 568.34 | 6.51 | 1 | [M+H-H_2_O]^+^ | 1.48 | RP-LC/MS | ESI + | 3 | 3.3 | 1.57 | - |
| ***Saccharides*** |  |  |  |  |  |  |  |  |  |  |  |  |
| Sucrose monopalmitate | C_28_H_54_O_13_ | 598.3564 | 6.09 | 2 | [M+K]^+^ | 1.90 | RP-LC/MS | ESI + | 3 | 0.4 | 1.47 | 3.79E-02 |
| ***Sphingolipids*** | |  |  |  |  |  |  |  |  |  |  |  |
| Erythro-Sphingosine C-15 | C_15_H_31_NO_2_ | 257.2355 | 9.05 | 5 | [M+H-H_2_O]^+^ | 1.53 | RP-LC/MS | ESI + | 3 | 3.1 | 1.81 | - |
| C17 Sphingosine | C_17_H_35_NO_2_ | 285.2668 | 10.5 | 5 | [M+H-H_2_O]^+^ | 4.62 | RP-LC/MS | ESI + | 3 | 4.3 | 1.84 | - |
| C19 Sphingosine-1-phosphate | C_19_H_40_NO_5_P | 393.2644 | 6 | 4 | [M+H-H_2_O]^+^ | 3.94 | RP-LC/MS | ESI + | 3 | 4.3 | 1.61 | 1.11E-02 |
| ***Sterol Lipids*** |  |  |  |  |  |  |  |  |  |  |  |  |
| Oxo-cholestenone | C_27_H_42_O_2_ | 398.3185 | 13.5 | 4 | [M+H]^+^ | 5.93 | RP-LC/MS | ESI + | 3 | 2.4 | 1.13 | - |
| Hydroxy-cholestenone | C_27_H_44_O_2_ | 400.3341 | 11.15 | 3 | [M+H]^+^ | 5.46 | RP-LC/MS | ESI + | 2 | 3.4 | 1.67 | 1.75E-02 |
| Hydroxy-norcholestatrienone | C_26_H_38_O_2_ | 382.2872 | 7.55 | 3 | [M+H]^+^ | 8.76 | RP-LC/MS | ESI + | 3 | 2.6 | 1.44 | 3.79E-02 |
| Methylenecholesterol | C_28_H_46_O | 398.3549 | 12 | 5 | [M+H]^+^ | 7.30 | RP-LC/MS | ESI + | 3 | 5.1 | 2.01 | - |
| ***Steroids*** |  |  |  |  |  |  |  |  |  |  |  |  |
| Dihydroxycholestenoic acid | C_27_H_44_O_4_ | 432.324 | 13.06 | 4 | [M+H]^+^ | 3.20 | RP-LC/MS | ESI + | 3 | 0.3 | 1.14 | - |
| Hydroxy-oxo-cholestenoate | C_27_H_42_O_4_ | 430.3083 | 12.39 | 1 | [M+H]^+^ | 5.67 | RP-LC/MS | ESI + | 3 | 0.1 | 1.20 | - |
| Dihydroxy-cholestenoate | C_27_H_44_O_4_ | 432.324 | 12.8 | 5 | [M+H]^+^ | 7.95 | RP-LC/MS | ESI + | 2 | 0.2 | 1.58 | 4.08E-03 |
| Oxo-cholenoic Acid | C_24_H_36_O_3_ | 372.2664 | 8.18 | 3 | [M+H]^+^ | 3.54 | RP-LC/MS | ESI + | 3 | 0.3 | 2.19 | 4.08E-03 |
| Hydroxy-oxocholenoic Acid | C_24_H_36_O_4_ | 388.2614 | 9.72 | 4 | [M+H]^+^ | 11.64 | RP-LC/MS | ESI + | 3 | 0.3 | 1.98 | 4.08E-03 |
| Epidioxy-cholestadienol | C_27_H_42_O_3_ | 414.3134 | 11.68 | 4 | [M+H]^+^ | 4.56 | RP-LC/MS | ESI + | 3 | 0.5 | 1.99 | 4.08E-03 |
| ^a^CV, coefficient of variation in the metabolites in the QC samples; ^b^Fold change in the abundance of the specified comparison calculated as (case/control); ^c^VIP, variable importance in projection; ^d^ p value < 0.05. RP: reversed-phase, HILIC: Hydrophilic interaction chromatography, LC: liquid chromatography, MS: mass spectrometry. | | | | | | | | | | | | |
